# The Pleiotropic Effects of Lipid-Modifying Interventions: Exploring Traditional and Emerging Hypolipidemic Therapies

**DOI:** 10.3390/metabo14070388

**Published:** 2024-07-17

**Authors:** Dimitris Kounatidis, Nikolaos Tentolouris, Natalia G. Vallianou, Iordanis Mourouzis, Irene Karampela, Theodora Stratigou, Eleni Rebelos, Marina Kouveletsou, Vasileios Stamatopoulos, Eleni Tsaroucha, Maria Dalamaga

**Affiliations:** 1 Diabetes Center, First Department of Propaedeutic Internal Medicine, Laiko General Hospital, Medical School, National and Kapodistrian University of Athens, 11527 Athens, Greece; dimitriskounatidis82@outlook.com (D.K.); ntentol@med.uoa.gr (N.T.); eleni.rebelos@utu.fi (E.R.); marinakouveletsou@gmail.com (M.K.); 2 First Department of Internal Medicine, Sismanogleio General Hospital, 15126 Athens, Greece; elenatsaroucha1@gmail.com; 3 Department of Pharmacology, National and Kapodistrian University of Athens, 11527 Athens, Greece; imour@med.uoa.gr; 4 Second Department of Critical Care, Attikon General University Hospital, Medical School, National and Kapodistrian University of Athens, 12462 Athens, Greece; eikaras1@gmail.com; 5 Department of Endocrinology and Metabolism, Evangelismos General Hospital, 10676 Athens, Greece; theodorastratigou@yahoo.gr; 6 Department of Internal Medicine, Evangelismos General Hospital, 10676 Athens, Greece; kypseli96@gmail.com; 7 Department of Biological Chemistry, Medical School, National and Kapodistrian University of Athens, 11527 Athens, Greece; madalamaga@med.uoa.gr

**Keywords:** ANGPTL3, bempedoic acid, cancer, CETP, ezetimibe, fibrates, inflammation, lipoprotein apheresis, recombinant HDL-C, PCSK9, pleiotropic properties, statins

## Abstract

Atherosclerotic cardiovascular disease poses a significant global health issue, with dyslipidemia standing out as a major risk factor. In recent decades, lipid-lowering therapies have evolved significantly, with statins emerging as the cornerstone treatment. These interventions play a crucial role in both primary and secondary prevention by effectively reducing cardiovascular risk through lipid profile enhancements. Beyond their primary lipid-lowering effects, extensive research indicates that these therapies exhibit pleiotropic actions, offering additional health benefits. These include anti-inflammatory properties, improvements in vascular health and glucose metabolism, and potential implications in cancer management. While statins and ezetimibe have been extensively studied, newer lipid-lowering agents also demonstrate similar pleiotropic effects, even in the absence of direct cardiovascular benefits. This narrative review explores the diverse pleiotropic properties of lipid-modifying therapies, emphasizing their non-lipid effects that contribute to reducing cardiovascular burden and exploring emerging benefits for non-cardiovascular conditions. Mechanistic insights into these actions are discussed alongside their potential therapeutic implications

## 1. Introduction

Dyslipidemia represents a significant global health challenge and is widely acknowledged as a pivotal risk factor for atherosclerotic cardiovascular disease (ASCVD), contributing to approximately 3.78 million deaths from ischemic heart disease (IHD) [1]. Over the years, a plethora of lipid-modifying therapies have been developed to address this critical issue, demonstrating efficacy in reducing not only lipid levels but also in exerting pleiotropic effects that extend beyond cardiovascular health.

Foremost among these therapies are statins, which inhibit 3-hydroxy-3-methylglutaryl-CoA reductase (HMGCR) to lower low-density lipoprotein cholesterol (LDL-C) levels. Their distinctive mechanism of action offers various other pleiotropic benefits, as evidenced in the literature. Statins are categorized by intensity, with rosuvastatin and atorvastatin recognized as the most potent [2] and by lipophilicity, influencing their cellular effects. Current evidence indicates that lipophilic statins exhibit more pleiotropic effects compared with hydrophilic statins because of their enhanced ability to penetrate cell membranes [3]. Ezetimibe complements statin treatment by reducing intestinal cholesterol absorption [4]. Recent guidelines advocate for the immediate initiation of ezetimibe as an adjunct to high-intensity statin therapy in individuals with ASCVD and LDL-C levels exceeding 110 mg/dL [5]. Both statins and ezetimibe have shown promise in mitigating inflammation and improving endothelial function impairment. However, statins are associated with diabetogenic effects with conflicting evidence regarding their role in malignancy, whereas the impact of ezetimibe on glucose metabolism and cancer risk remains a subject of debate [6,7].

In addition to the well-established benefits of traditional lipid-lowering therapies in reducing cardiovascular disease (CVD) risk, recent years have witnessed the emergence of novel agents. Proprotein convertase subtilisin/kexin type 9 (PCSK9) inhibitors, such as monoclonal antibodies evolocumab and alirocumab, along with the synthesis inhibitor inclisiran, have significantly advanced hypercholesterolemia treatment. PCSK9 inhibition increases LDL receptor (LDL-R) density in liver cells, enhancing LDL-C particle clearance and markedly lowering LDL-C levels [8]. Another promising agent, bempedoic acid (BA), has emerged as a prominent therapeutic for managing dyslipidemia, particularly in cases of statin intolerance. BA inhibits cholesterol biosynthesis by targeting adenosine triphosphate-citrate lyase (ACLY), an enzyme upstream of HMGCR. As a pro-drug, BA is primarily converted into its active metabolite by very-long-chain acyl-CoA synthetase 1, predominantly found in the liver, thereby mitigating the risk of myopathy [9]. Evidence for both PCSK9 inhibitors and BA suggests encouraging effects on inflammation and malignancy. However, PCSK9 inhibitors may lead to mild hyperglycemia, although evidence is conflicting [10,11]. On the contrary, BA shows promise in enhancing insulin sensitivity [12].

In addition to traditional and novel agents for dyslipidemia treatment, recent years have seen the introduction of alternative therapeutic approaches, particularly for refractory cases of familial hypercholesterolemia (FH). Two notable agents in this field are mipomersen and lomitapide. Mipomersen, a second-generation antisense oligonucleotide, selectively degrades apolipoprotein B100 (ApoB100) mRNA [13]. Lomitapide, an oral inhibitor of microsomal triglyceride transfer protein (MTP), primarily affects very low-density lipoprotein cholesterol (VLDL) production containing ApoB100. Despite their initial promising lipid-lowering efficacy, their use is now limited because of concerns regarding hepatotoxicity [13,14]. Therefore, mipomersen and lomitapide have not been extensively investigated for their pleiotropic effects, although recent experimental studies note the potential beneficial effects of lomitapide in both inflammation and malignancy [15].

In 2018, the Food and Drug Administration (FDA) approved a novel class of cholesterol-lowering medications known as angiopoietin-like 3 protein (ANGPTL3) inhibitors, namely evinacumab and vupanorsen. These innovative compounds target ANGPTL3, enhancing lipoprotein lipase (LPL) and endothelial lipase (EL) activities. This mechanism results in significant LDL-C reductions independent of the LDL-R activity [16]. Beyond their impact on lipid-lowering, several studies have demonstrated their potential benefits in inflammation and cancer [17]. Additionally, individuals with familial hypercholesterolemia may benefit from lipoprotein apheresis (LA), a therapeutic modality designed to selectively eliminate ApoB-containing lipoproteins from circulation [18]. Despite its questionable role in mitigating CVD risk, LA has demonstrated impressive pleiotropic benefits, including anti-inflammatory effects and improvements in vascular health [19].

In the last few years, novel pharmaceutical agents have emerged that enhance the levels of high-density lipoprotein cholesterol (HDL-C). These agents work either by mimicking the properties of natural HDL-C particles (recombinant HDL-C particles) or by inhibiting cholesteryl ester transfer protein (CETP). CETP, a glycoprotein primarily produced by the liver, facilitates the transfer of cholesteryl esters from HDL-C to VLDL, chylomicrons, and LDL-C while simultaneously transferring triglycerides (TGs) from these particles to HDL-C. Although these drugs significantly increase HDL-C concentrations, they have not delivered the anticipated cardiovascular benefits. Nevertheless, their ability to enhance HDL-C and its associated apolipoproteins has prompted further investigations into their broader impacts beyond traditional lipid management. Emerging evidence from preclinical and clinical studies supports the beneficial effects of CETP inhibitors and recombinant HDL-C (rHDL) particles in treating inflammatory diseases, improving glucose metabolism, and potentially combating certain cancers [20,21]. Similarly, niacin, a potent HDL-C-raising drug with a favorable impact on lowering LDL-C and TGs, has shown disappointing results in reducing CVD risk but exhibits diverse pleiotropic effects [22].

Another area of interest lies in elucidating the pleiotropic properties of agents that ameliorate hypertriglyceridemia, particularly peroxisome proliferator-activated receptor alpha (PPARa) agonists such as fibrates. Clinical studies have shown that fibrates, such as fenofibrate, offer benefits by improving endothelial function, mitigating inflammation, and enhancing insulin sensitivity. Moreover, they hold promise in cancer therapy through their influence on various pathways involved in tumor suppression and apoptosis [23]. Beyond fibrates, the management of hypertriglyceridemia includes the prescription of omega-3 fatty acids (FAs), typically as supplementary treatment, and the use of apolipoprotein CIII (ApoCIII) inhibitors, particularly Volanesorsen, especially in severe cases. Both treatment approaches have shown promising pleiotropic effects, with a reduction in inflammation being central to these benefits [24,25]. 

This review aims to delve into the diverse pleiotropic effects of lipid-modifying agents beyond their primary role in lowering lipid levels, emphasizing their potential implications in various health issues, with a focus on inflammation, vascular function, glucose metabolism, and malignancy.

## 2. Literature Search Methodology

For this narrative review, we systematically searched the PubMed database using a comprehensive array of keywords related to lipid-lowering therapies and their mechanisms. Specifically, our search terms encompassed “statins”, “ezetimibe”, “PCSK9 inhibitors”, “bempedoic acid”, “mipomersen”, “lomitapide”, “ANGPTL3 inhibitors”, “lipoprotein apheresis”, “CETP inhibitors”, “recombinant HDL-C”, “niacin”, “fibrates”, “omega-3 fatty acids”, “ApoCIII inhibitors”, and “pleiotropic properties”. Our primary objective was to compile pertinent research articles, randomized clinical trials, and meta-analyses. Moreover, we meticulously examined the references cited within these articles to identify the relevant supplementary literature. It is acknowledged, however, that due to the substantial volume of retrieved manuscripts, this review may not encompass the entirety of the available literature on the subject.

## 3. Traditional and Novel Lipid-Modifying Treatments That Primary Reduce LDL-C

### 3.1. Statins

Statins are pivotal in the management of hyperlipidemia, leveraging their unique mechanism of inhibiting HMGCR. This action primarily targets a reduction in ApoB-containing lipoproteins, notably LDL-C. Statins achieve this by either curbing ApoB synthesis in the liver or boosting the clearance of LDL through increased expression of LDL-R on liver cells [26]. Rosuvastatin, the most potent statin at a maximal dose of 40 mg, can reduce LDL-C by up to 55%, lower TGs by 25%, and increase HDL-C by up to 8% [27]. Currently, statins remain the cornerstone of lipid-lowering treatment, offering numerous benefits beyond cardiovascular health as they demonstrate a wide range of pleiotropic effects [26]. 

#### 3.1.1. Inflammation

In addition to their lipid-lowering effects, the inhibition of HMGCR by statins also augments their anti-inflammatory properties. HMGCR activation has been reported to initiate inflammatory responses through various pathways. Typically, HMGCR upregulates Toll-like receptors (TLRs), thereby elevating levels of pro-inflammatory mediators and leukocyte adhesion molecules [28]. This upregulation further boosts major histocompatibility complex II (MHC)-II expression, prompting T-cell activation and facilitating the secretion of pro-inflammatory cytokines such as interleukin-1 (IL-1), interleukin-6 (IL-6), and tumor necrosis factor-alpha (TNF-α) [29]. Consequently, inhibiting HMGCR via statins diminishes TLR4 expression on immune cells, a pivotal regulator of the NLR family pyrin domain containing 3 (NLRP3) inflammasome. Recent research has spotlighted the NLRP3 inflammasome and its downstream mediators as potential targets for statins [30]. 

The antioxidant benefits of statins, propelled by HMGCR, have also been extensively documented and are linked to their capacity to enhance crucial antioxidant enzymes via the phosphatidylinositol 3-kinase/protein kinase B (PI3K/Akt) pathway, consequently curtailing the generation of reactive oxygen species (ROS) [31]. Meta-analyses have underscored that statin therapy bolsters antioxidant properties by markedly elevated circulating levels of glutathione peroxidase (GPx) and superoxide dismutase (SOD) [32]. 

Moreover, statins are recognized for their prowess in scavenging oxygen free radicals, a function associated with the stabilization of the nuclear factor kappa-light-chain-enhancer of activated B cells (NF-κB) inhibitor protein, IκBα [33]. Evidence also indicates that statins curb the production of inflammatory cytokines by downregulating the transcription factor NF-κB within the mevalonate pathway [34]. Last, recent research has highlighted the potential role of statins in inducing ferroptosis through the mevalonate pathway, which is critical for synthesizing GPx4 and forming coenzyme Q10 (CoQ10) [35]. Moreover, they inhibit pyroptosis, a form of programmed cell death associated with inflammation, by blocking the long noncoding RNA nexilin F-actin binding protein antisense RNA 1/nexilin F-actin binding protein (lncRNA NEXNAS1/NEXN) pathway, reducing the release of pro-inflammatory cytokines such as IL-1 and IL-18 [36]. 

Numerous studies have also confirmed the effectiveness of statins in reducing C-reactive protein (CRP) and high-sensitivity CRP (hs-CRP) levels. CRP is an inflammatory marker indicative of CVD risk, with statins reportedly lowering CRP levels by up to 60% [37]. A recent meta-analysis corroborated the beneficial effects of statins on reducing CRP and hs-CRP levels, independent of the statin’s intensity or the type of CVD. This analysis found that treatment durations exceeding 10 weeks led to decreased hs-CRP levels, although only high-intensity statin regimens marginally reduced CRP serum concentrations in individuals with CVD [38]. Additionally, another meta-analysis provided guidance for selecting statin therapy based on LDL-C and CRP levels, concluding that simvastatin at 40 mg/day was the most effective, while atorvastatin at 80 mg/day offered the best long-term outcomes [39]. 

Clinically, the anti-inflammatory effects of statins facilitated through HMGCR inhibition have proven advantageous in treating various conditions, including Alzheimer’s disease. HMGCR assumes a pivotal role in the development of neuroinflammation [40] while also provoking microglial proliferation, which can lead to synaptic damage and neuronal apoptosis, primarily via activation of the NF-κB signaling pathway [41]. Additionally, HMGCR facilitates the deposition of amyloid-beta (Aβ), thereby contributing to cognitive deficits [42]. As for Parkinson’s disease (PD), the literature presents conflicting evidence. While some studies suggest that statins exert a positive impact by regulating inflammatory and lysosomal signaling pathways, others argue that they may elevate PD risk by reducing levels of the antioxidant CoQ10 [43].

Statins have demonstrated anti-inflammatory benefits in individuals with chronic kidney disease (CKD) as well. A recent meta-analysis revealed significant anti-inflammatory properties associated with statin use, leading to improvements in complications, slowing CKD progression, and reducing mortality rates. Notably, subgroup analyses indicated a substantial reduction in CRP levels among both predialysis CKD and dialysis patients receiving short- or long-term statin therapy [44]. Moreover, there is emerging evidence suggesting potential benefits of statin treatment for individuals with inflammatory bowel disease (IBD), attributed to statins’ immunomodulatory properties, which include inhibiting T-cell activation, antigen-presenting function, and leukocyte tissue infiltration [45]. A population-based case-control study conducted in Sweden found that statin prescription might be associated with a decreased risk of Crohn’s disease, although not ulcerative colitis [46]. 

#### 3.1.2. Vascular Health

Statins have the potential to improve endothelial function by inhibiting the NLRP3 inflammasome. Specifically, they may reduce the activation of the NLRP3 inflammasome triggered by oxidized LDL (OxLDL) or TNFα in vascular endothelial cells via a mechanism dependent on the pregnane X receptor (PXR) [47]. PXR is a nuclear receptor that regulates the expression of genes involved in drug metabolism and transport, thereby aiding in the detoxification and elimination of xenobiotics and endotoxins from the body [48]. Furthermore, statins are widely recognized for their beneficial impact on vascular health, partly because of their ability to modulate pro-oxidant enzymes and enhance endothelial nitric oxide synthase (eNOS) functionality. This modulation includes mitigating the activity of Nicotinamide Adenine Dinucleotide Phosphate (NADPH) oxidase, a primary source of ROS in the vasculature, and promoting eNOS expression, activity, and enzymatic coupling [49,50]. Current evidence supports that statins can reduce NADPH oxidase activity in both endothelial cells and vascular smooth muscle cells, thereby decreasing ROS production [51]. Additionally, statins enhance eNOS activity by increasing its phosphorylation at specific activation sites, which in turn boosts nitric oxide (NO) availability and improves endothelium-dependent vasorelaxation [52]. Statins have also been shown to elevate heme oxygenase-1 (HO-1) activity through p38 and PI3K/Akt-dependent mechanisms. HO-1 is a critical factor with antioxidative, anti-inflammatory, anti-proliferative, and anti-apoptotic properties in the vasculature [53,54]. 

The antithrombotic properties of statins are largely due to their ability to inhibit platelet activation and exert anticoagulant effects [55]. Statins achieve this by upregulating eNOS and downregulating cyclooxygenase-1 activation, thereby inhibiting platelet activation [56]. They also reduce tissue factor (TF) activity by downregulating geranylgeranylated proteins, which play a significant role in reducing TF [57]. Furthermore, statins enhance thrombomodulin mRNA levels in a dose-dependent manner by inhibiting the geranylgeranylation of Rho subfamily proteins, such as NF-κB, ultimately activating the protein C pathway and exhibiting anticoagulant activity [58]. A meta-analysis by Kunutsor et al. found that statin use is associated with a significant reduction in deep vein thrombosis risk, with rosuvastatin showing the lowest risk among statins [59]. 

Recent research has also highlighted the epigenetic modulatory effects of statins, particularly in preventing endothelial-to-mesenchymal transition, which is a process linked to endothelial dysfunction. Simvastatin has been shown to significantly improve the function of human-induced pluripotent stem cell-derived endothelial cells under both normal and diabetic conditions [60].

#### 3.1.3. Glucose Metabolism

Despite their groundbreaking role in mitigating CVD risk, statins have been associated with insulin resistance and the development of new-onset diabetes mellitus (NODM). It is known that statin use can elevate fasting glucose and insulin levels and increase insulin resistance, particularly in a dose-dependent manner [61]. Notably, these adverse effects are more commonly observed with high-intensity statin therapy [6]. The molecular mechanisms underlying statin-induced NODM are not fully understood, although several have been proposed. One mechanism involves the inhibition of the HMGCR receptor and the disruption of intracellular calcium (Ca^2+^) concentration, primarily regulated by calcium channels, which can significantly impair glucose homeostasis [62].

Statins have also been shown to induce insulin resistance by reducing the translocation of glucose transporter type 4 (GLUT4) to the cell membrane, thereby decreasing peripheral insulin-mediated glucose uptake [63]. Additionally, statins may lower adiponectin levels in adipose tissue, diminishing its beneficial effects on reducing hepatic gluconeogenesis and its protective and regenerative effects on pancreatic beta cells [64]. Recently, Henriksbo et al. have identified p38 and mammalian target of rapamycin (mTOR) as mediators of statin-induced insulin resistance in adipose tissue, demonstrating the ability of statins to activate the NLRP3 inflammasome [65]. Finally, statins might influence genetic and epigenetic mechanisms, including changes in microRNAs, which are known to have diabetogenic effects due to their impact on gene expression in dysfunctional beta cells and insulin-resistant tissues [62].

#### 3.1.4. Malignancy

The anti-cancer benefits of statins have been acknowledged since the 1990s, though they remain a topic of debate. Findings from epidemiological and clinical studies on the relationship between statins and cancer are mixed. A large 15-year observational study in Denmark found reduced mortality for 13 types of cancer among statin users compared with non-users, although it did not find a dose-dependent relationship [66]. Certain studies have shown the benefits of statin use in specific populations, such as postmenopausal women and men with prostate cancer [67]. The duration of statin use also appears to have varying effects on different cancers. For instance, a Japanese study of 67,768 participants found that using statins for more than five years was associated with a reduced risk of liver cancer but an increased risk of pancreatic cancer [68]. Conversely, an 8.8-year Finnish study involving nearly 900,000 people found no link between statin use and cancer [69].

Meta-analyses also offer conflicting results regarding the anti-cancer effects of statins. One meta-analysis involving over a million cancer patients suggested that statin use could reduce cancer-specific mortality by 40% [70]. In contrast, another meta-analysis of 27 clinical trials with 175,000 patients found no evidence that a five-year statin prescription reduced cancer prevalence or mortality [71]. Furthermore, long-term studies exceeding five years did not demonstrate a significant association between statin use and the incidence of overall or specific cancers [72].

The mechanisms through which statins may exert anti-cancer effects have been demonstrated in both in vitro and in vivo studies. In vitro evidence indicates that statins inhibit HMGCG, which is essential for regulating metalloproteinase synthesis through its influence on geranylgeranyl pyrophosphate (GGPP) production [73]. Statins such as simvastatin and fluvastatin reduce matrix metalloproteinase (MMP) 9 release in murine and human macrophages [74]. In vivo evidence indicates that simvastatin may reduce bone metastasis and tumor growth in a mouse model of human lung cancer xenograft by decreasing mitogen-activated protein kinase/extracellular signal-regulated kinase (MAPK/ERK) activity [75]. Simvastatin also promotes eNOS phosphorylation, prevents apoptosis, and enhances angiogenesis via an Akt-dependent mechanism [76]. Pitavastatin can inhibit subcutaneous glioma cell growth [77], while the combination of fluvastatin with gemcitabine can delay and weaken tumor growth in a pancreatic cancer xenograft [78]. Notably, lipophilic statins appear to be more effective in treating cancer than hydrophilic statins because of their higher proapoptotic and cytotoxic potential [79].

### 3.2. Ezetimibe

Ezetimibe, an azetidine derivative, selectively inhibits Niemann-Pick C1-like protein 1 (NPC1L1), which blocks intestinal cholesterol absorption. This action reduces the incorporation of cholesterol into chylomicrons and subsequently decreases the intrahepatic cholesterol pool. As a result, LDL-R expression is upregulated, enhancing the clearance of ApoB100-containing lipoproteins from the plasma [80]. In clinical settings, ezetimibe is often used as an adjunct to statins for patients who do not reach their LDL-C targets with optimal statin therapy alone. Notably, the combination of ezetimibe with a high-intensity statin can lead to a significant reduction in LDL-C levels, potentially achieving a decrease of around 70% [81].

#### 3.2.1. Inflammation

Beyond its notable efficacy in reducing LDL-C levels, ezetimibe’s anti-inflammatory properties have garnered recognition for several years, prompting numerous studies to investigate the potential underlying mechanisms of action. The influence of ezetimibe on CRP levels has sparked ongoing discourse within the scientific community, particularly in its role as an adjunct therapy for individuals undergoing statin treatment. A comprehensive analysis conducted by Pearson et al. revealed that ezetimibe administration led to a notable 6% decrease in CRP levels compared with a placebo. Furthermore, the addition of ezetimibe to statin therapy exhibited a substantial supplementary reduction in CRP, with a treatment disparity of 10% [82]. In a meta-analysis conducted by the same researchers, the impact of simvastatin monotherapy was juxtaposed with combined ezetimibe/simvastatin treatment regarding CRP levels. The results indicated that ezetimibe/simvastatin combination therapy induced significantly greater reductions in CRP when compared with simvastatin alone, showcasing reductions of 31.0% versus 14.3%, respectively, even at varying simvastatin doses [83]. 

However, conflicting results emerged from a study conducted by Oh et al., which demonstrated that in hypercholesterolemic subjects, co-administration of ezetimibe and statins failed to yield significant reductions in CRP levels, despite a marked decrease in LDL-C concentrations [84]. These observations were echoed by a meta-analysis encompassing 23 controlled trials, which found no significant advantage in incorporating ezetimibe alongside maximal statin therapy concerning IL-6 levels, the primary driver of CRP synthesis. Nevertheless, this same meta-analysis highlighted that ezetimibe/statin co-administration enhanced TNF-α-lowering effects compared with statin monotherapy [85]. These contrasting findings suggest that the anti-inflammatory effects of ezetimibe may be mediated through mechanisms independent of LDL-C reduction. This hypothesis gains further credence from a recent meta-analysis encompassing 171,668 subjects from 53 randomized controlled trials (RCTs). This study scrutinized the impact of various lipid-lowering agents on both LDL-C and CRP levels and revealed that ezetimibe elicited a significant 28% reduction in CRP levels, irrespective of LDL-C modifications [86].

Exploring alternative potential mechanisms, independent of LDL-C, through which ezetimibe exerts its anti-inflammatory role suggests that the drug might achieve this effect by engaging various intracellular signaling pathways. In particular, diminished NF-κB activation has been reported upon promoting IκB degradation through the MAPK pathway [87]. This modulation of intracellular signaling pathways by ezetimibe suggests emerging favorable outcomes regarding certain pathological conditions. In ischemic stroke, ezetimibe demonstrates potential benefits by reducing inflammation and oxidative stress via the adenosine monophosphate-activated protein kinase(AMPK)/Nrf2/TXNIP pathway, as observed in an experimental model of middle cerebral artery occlusion [88]. 

Additionally, preliminary evidence indicates that ezetimibe may offer advantages in treating steatohepatitis by inducing autophagy, an effect mediated by AMPK activation and inhibition of the NLRP3 inflammasome [89]. Furthermore, ezetimibe has been reported to exhibit anti-inflammatory properties in individuals with chronic inflammatory conditions, such as ankylosing spondylitis (AS). In vitro data indicate that ezetimibe could serve as an effective therapy for AS patients by mitigating the expression of Th17 differentiation-related genes, including IL-23R and IL-1R [90]. 

#### 3.2.2. Vascular Health

The impact of ezetimibe on endothelial function has been debated, with conflicting evidence from both experimental and clinical studies. Several clinical trials have not shown significant benefits of ezetimibe, even as an add-on to statin therapy, and its lack of pleiotropic efficacy may be understood in the context of its pharmacokinetic profile and mechanism of action. Ezetimibe is rapidly absorbed, with about 80% of the administered dose eliminated in the feces, resulting in decreased systemic exposure [91,92,93]. 

Experimental studies suggest that ezetimibe administration can enhance endothelial function in atherosclerotic mouse models. Ezetimibe may improve acetylcholine-mediated vasodilatory responses and boost eNOS expression, thereby positively impacting cytokine expression and oxidative stress [94]. Furthermore, ezetimibe may exhibit antioxidant and anti-thrombotic properties by reducing platelet aggregation, urokinase-type plasminogen activator expression, and LDL-C peroxidation in endothelial cells [95,96]. 

Similar beneficial effects have been reported in the clinical setting. In cases where ezetimibe improves endothelial function, the effect is primarily attributed to its significant cholesterol-lowering ability rather than the direct cellular effects seen with statin therapy [97]. The lipid-lowering properties of ezetimibe have been associated with improvements in endothelial function. Ezetimibe monotherapy ameliorates postprandial hyperlipidemia, which is closely linked to transient endothelial dysfunction, as evidenced by increased brachial artery flow-mediated dilation (FMD) [98]. Additionally, in obese subjects with metabolic syndrome and coronary artery disease (CAD), ezetimibe improves postprandial hyperinsulinemia, thereby positively impacting endothelial function [99]. 

When ezetimibe is co-administered with statins, it demonstrates notable benefits for endothelial function. The CuVIC trial highlighted that the adjunctive administration of ezetimibe to statin therapy improved endothelial function in stented coronary arteries more effectively than statin monotherapy, attributed to greater reductions in OxLDLs and oxysterol levels [100]. Notably, in patients with hypercholesterolemia, heart failure, and CAD, ezetimibe-containing regimens seem to be less effective than statin monotherapy for improving endothelial function when both treatments achieve equivalent cholesterol reductions [97]. 

In conclusion, current evidence suggests that ezetimibe monotherapy is less effective than statin monotherapy in improving endothelial function [101]. However, a low-dose statin combined with ezetimibe may offer comparable or superior benefits to high-dose statin therapy alone [102,103]. 

#### 3.2.3. Glucose Metabolism

The effects of ezetimibe on glucose metabolism are conflicting. Some studies report that ezetimibe improves insulin resistance and reduces visceral fat, while others suggest that it may cause hyperglycemia. Various mechanisms might explain the beneficial effects of ezetimibe, particularly in individuals with diabetes mellitus type 2 (T2DM). One notable mechanism is the increased expression of NPC1L1 mRNA in the duodenum of T2DM patients since NPC1L1 is the primary target of ezetimibe [104]. Experimental evidence in high-fat diet-fed diabetic mouse models shows that ezetimibe may enhance active glucagon-like peptide-1 levels in the intestine, reduce adipocyte size in visceral fat, lower serum levels of free fatty acids, induce fatty acid oxidation, improve adipocytic inflammation, and partially improve glycemic index values [105]. 

Research has also explored the impact of combining ezetimibe with statins on insulin sensitivity in T2DM patients [106]. It is suggested that replacing high-dose statin treatment with ezetimibe, either alone or combined with low-dose statins, may reduce the risk of hyperglycemia. A meta-analysis of 16 RCTs found that add-on ezetimibe therapy in patients on low-dose statins for over three months significantly reduced fasting glucose levels compared with high-dose statin therapy [107]. However, recent evidence indicates that while the rosuvastatin/ezetimibe combination therapy is more effective at reducing LDL-C levels than rosuvastatin alone, it does not significantly enhance insulin sensitivity or reduce vascular inflammatory responses. Markers of insulin resistance and oxidative stress (HOMA-IR and PRDX4) did not differ significantly between the two groups after 12 weeks of treatment [108].

#### 3.2.4. Malignancy

The combination of ezetimibe and statins has been associated with a heightened malignancy risk [7]. Nevertheless, recent years have seen a surge in preclinical evidence suggesting that ezetimibe could confer advantages in the onset and advancement of diverse cancer types, including breast cancer [109], gastrointestinal tumors [108], and renal cell carcinoma [110]. These positive anti-cancer outcomes of ezetimibe are believed to stem from its anti-inflammatory characteristics, as well as its beneficial effects on stem cell suppression, cellular proliferation, immune modulation, and angiogenesis [108]. 

In an experimental mouse model of hypercholesterolemic urinary bladder cancer, ezetimibe decreased the proportion of cancer cells expressing CK5, CK14, and p-STAT3, as well as cancer stemness markers such as ALDH1A1 and CD44 [111]. Furthermore, recent data support that ezetimibe promotes antitumor immunity and mitigates prostate tumor growth and metastasis by inhibiting protein kinase B (Akt) phosphorylation and mammalian target of rapamycin complex 2 (mTORC2) signaling in lymphocytes [110]. Notably, in models of steatohepatitis-related hepatocellular carcinoma, ezetimibe exhibits anti-cancer properties by lowering serum and liver cholesterol levels and inhibiting angiogenesis induced by CD31 and vascular endothelial growth factor (VEGF) [112]. 

### 3.3. PCSK9 Inhibitors

Proprotein convertase subtilisin/kexin type 9 (PCSK9) is a serine protease known for its role in regulating LDL-R levels in hepatocytes [113]. PCSK9 inhibitors, such as monoclonal antibodies such as evolocumab and alirocumab, as well as synthesis inhibitors such as inclisiran, form a diverse group of medications primarily prescribed for treating primary hypercholesterolemia or mixed dyslipidemia. These inhibitors function by increasing LDL-R density in hepatocytes, which enhances the clearance of LDL-C particles and reduces plasma LDL-C levels [114]. Clinical trials consistently show that PCSK9 inhibition, when added to maximally tolerated statin treatment, reduces serum LDL-C levels by 50 to 65%, regardless of the specific agent or dosage regimen used [115]. Despite their relatively recent introduction, numerous studies have also highlighted the anti-inflammatory benefits of these agents.

#### 3.3.1. Inflammation

Traditional LDL-C-lowering agents, particularly statins, have been proven to reduce hsCRP levels notably. However, randomized clinical trials involving PCSK9 inhibitors have not shown a similar effect. In the FOURIER trial, which included 27,564 patients with stable ASCVD and LDL-C levels over 70 mg/dL despite statin treatment, participants were given either evolocumab or a placebo. Over a median period of 2.2 years, the trial assessed evolocumab’s impact on cardiovascular outcomes, including cardiovascular death, myocardial infarction, stroke, hospitalization for unstable angina, or coronary revascularization. The analysis, stratified by baseline hsCRP levels, demonstrated that evolocumab reduced CVD events across all hsCRP levels, with more significant reductions seen in those with higher baseline hsCRP levels [116]. In contrast, a meta-analysis from five years ago involving 4,198 participants found no significant effect of PCSK9 monoclonal antibodies (PCSK9-mAbs) on circulating hsCRP levels. This finding remained consistent across participant characteristics, PCSK9-mAb types, determination methods, and treatment duration [117]. 

In an observational study involving 645 patients who had been on stable therapy for at least six months and were undergoing carotid endarterectomy, researchers examined the effects of PCSK9 inhibitors on the expression of various inflammation-associated factors within atheromatous plaques. This study revealed that patients treated with PCSK9 inhibitors exhibited reduced expression of pro-inflammatory proteins, such as IL-1β and TNFα, despite similar levels of circulating hs-CRP. This effect was consistent even in subgroups with LDL-C levels below 100 mg/dL, suggesting an independent anti-inflammatory action of PCSK9 inhibitors [118]. As for inclisiran, it appears to exert its anti-inflammatory properties by inhibiting IL-1α, IL-6, and TNF-α in OxLDL-stimulated THP-1-derived macrophages through the suppression of NF-κB nuclear translocation [119]. Additionally, although not statistically significant for individual doses, inclisiran at 300 mg and 500 mg may lead to a reduction in hs-CRP levels (16.2% and 19.8%, respectively) [120]. 

In recent years, several experimental studies have illuminated the potential anti-inflammatory capacity of PCSK9 inhibition by regulating specific intracellular signaling pathways. A growing body of evidence indicates an interplay between PCSK9 expression and the activation of NLRP3 inflammasome signaling, emphasizing NLRP3 inhibition as a prospective therapeutic target of PCSK9 inhibition. Specifically, PCSK9 governs caspase-1-dependent pyroptosis by instigating mitochondrial DNA damage and activating NLRP3 inflammasome signaling, while the NLRP3 inflammasome, in turn, exerts its influence via IL-1β to regulate PCSK9 secretion [121,122,123]. Recently, Shin et al. have unveiled the PCSK9-CAP1-Syk/PKCδ pathway as a potential novel target of PCSK9 inhibitor activity. Their study outcomes revealed that PCSK9 might contribute to atherosclerosis by inducing NF-κB and inflammatory genes in monocytes, independent of LDL-R involvement within this experimental framework, PCSK9 bonded to cyclase-associated actin cytoskeleton regulatory protein 1 (CAP1), its primary binding partner, which is crucial for the inflammatory characteristics of PCSK9, thereby leading to escalated cytokine production and the activation of TLR4 and scavenger receptors, thereby fostering the uptake of OxLDLs by macrophages [124]. 

#### 3.3.2. Vascular Health

Mounting evidence highlights the connection between serum PCSK9 and endothelial dysfunction, shedding light on the favorable outcomes associated with PCSK9 inhibition [125]. The precise mechanism through which PCSK9 inhibitors enhance endothelial function remains a subject of ongoing research. However, existing insights suggest that this positive impact could stem from a reduction in LDL-C levels or the mitigation of PCSK9’s capacity to incite a macrophage-mediated inflammatory response. PCSK9’s role in upregulating VEGF-A and intercellular adhesion molecule (ICAM-1) expression promotes endothelial cell activation and facilitates monocyte/macrophage migration, thereby fostering an inflammatory milieu conducive to atherosclerosis [126]. Both in vivo and ex vivo data indicate that PCSK9 inhibitors may mitigate endothelial dysfunction by suppressing endothelial chemokine production and reducing leukocyte-endothelium interactions [127]. 

Additionally, research suggests that PCSK9 monoclonal antibodies may influence circulating endothelial progenitor cells [128], crucial players in vascular repair following endothelial injury [129]. Experimental sepsis models have shown promising results, indicating that PCSK9 inhibitors counteract sepsis-induced inflammation and endothelial dysfunction driven by heightened PCSK9 expression, mediated through the TLR4/MyD88/NF-κB and NLRP3 pathways [130]. Furthermore, PCSK9 silencing appears to mitigate endothelial cell apoptosis via the MAPK signaling pathway [131]. Clinical studies corroborate these findings, demonstrating that a two-month treatment with evolocumab at 140 mg can improve endothelial function in individuals at elevated cardiovascular risk, as evidenced by enhancements in mean brachial artery diameter, velocity time integral, and FMD [132]. 

Recent knowledge has underscored the potential of PCSK9 to influence platelet function, suggesting that its inhibition might mitigate this effect [133]. In vivo research indicates that the pro-thrombotic properties of PCSK9 are primarily linked to its interaction with the CD36 receptor on platelets. This receptor recognizes specific oxidized phospholipids and lipoproteins, initiating signaling pathways that promote platelet activation and thrombosis [134]. Serum PCSK9 interferes with the CD36 receptor, leading to the activation of ROS-upregulating enzymes such as Src kinase, MAPK, extracellular signal-regulated kinase 5, and c-Jun N-terminal kinase [135]. 

Interestingly, recent data suggest that platelets might produce PCSK9, creating a feedback loop that enhances platelet aggregation and thrombus formation [136]. Notably, the anti-thrombotic properties of PCSK9 inhibitors could partly be due to their ability to lower serum lipoprotein(a) [Lp(a)] levels, which has been associated with a 31% reduction in the relative risk of venous thromboembolism [137,138]. Last, PCSK9 inhibition may offer anti-thrombotic benefits because of its interaction with factors related to coagulopathy. Specifically, PCSK9 inhibitor treatment may lead to a reduction in plasminogen activator inhibitor-1 (PAI-1) levels, resulting in enhanced fibrinolytic activity [139]. Additionally, PCSK9 silencing may positively impact the levels of TF, further contributing to its anti-thrombotic effects [140]. 

#### 3.3.3. Glucose Metabolism

Safety outcomes from significant cardiovascular trials, such as the ODYSSEY trial for alirocumab [141] and the FOURIER trial for evolocumab [142], have not indicated an increased occurrence of NODM. Similarly, meta-analyses have not identified a heightened risk of NODM in patients treated with PCSK9 inhibitors. For instance, a meta-analysis of RCTs assessing the effects of statins and statins combined with PCSK9 inhibitors in non-diabetic participants assigned to either more intensive or less intensive lipid-lowering therapies revealed that neither LDL-C reduction nor the use of PCSK9 inhibitors was associated with the onset of NODM [143]. 

Conversely, Mendelian randomization data have shown an increased likelihood of developing NODM among individuals with genetic variants at the PCSK9 locus. Supporting this, Mbikay et al. have found that PCSK9-deficient mice demonstrate impaired glucose tolerance and altered glucose-stimulated insulin secretion, mainly due to reduced insulin secretion rather than peripheral insulin resistance [144]. These results are consistent with post-marketing safety data, indicating that PCSK9 monoclonal antibodies may be linked to mild hyperglycemia, particularly within the first six months of treatment [145]. 

#### 3.3.4. Malignancy

PCSK9 overexpression has been identified in various solid tumors and hematological malignancies [146], including breast cancer, hepatocellular carcinoma, colorectal cancer, and lymphoblastic leukemia [147,148,149,150]. Elevated PCSK9 levels are correlated with increased cancer severity and poorer prognosis, whereas lower PCSK9 levels are associated with improved outcomes and reduced tumor growth. These observations have led to recent research focusing on the potential of PCSK9 inhibition to debilitate malignancy progression.

Experimental models, both in vitro and in vivo, have elucidated several mechanisms by which PCSK9 inhibition may promote apoptosis in cancer cells. Specifically, PCSK9 inhibition can interfere with the progression of the cell cycle, inhibiting cell proliferation and inducing endoplasmic reticulum (ER) stress, leading to apoptotic cell death. Furthermore, PCSK9 inhibition affects mitochondrial pathways by activating pro-apoptotic molecules such as caspase-3 and downregulating anti-apoptotic proteins such as phosphorylated Akt (p-Akt) and survivin [151]. Additionally, PCSK9 inhibitors can downregulate pathways crucial for cancer progression, including the MAPK pathway, the KRAS/MEK/ERK signaling pathway, and the Jak2/STAT3 signaling pathway [152,153].

The anti-cancer potential of cholesterol depletion through PCSK9 inhibition represents another mechanism of action against tumor progression. A high-cholesterol diet was shown to reverse the protective effects of PCSK9 knockout against melanoma metastasis in the liver [154]. Recent findings additionally underscore the role of PCSK9 inhibition in bolstering the antitumor immune response. Specifically, reducing PCSK9 levels may result in heightened expression of MHC-I on tumor cell surfaces [155] and augmentation of CD8+ T cell infiltration within tumors [156]. This increase in MHC-I expression hinders cancer cells from evading immune detection [157], while the rise in CD8+ T cell infiltration suggests decreased cancer proliferation and a lowered likelihood of metastasis and recurrence [158].

However, the impact of PCSK9 inhibition may vary across different cancers. For instance, anti-PCSK9 vaccination in mice with melanoma tumors did not result in reduced tumor growth or improved survival outcomes [159]. Notably, PCSK9 siRNA was reported to protect prostate cancer cells from ionizing radiation-induced damage because of its anti-apoptotic properties [160]. Moreover, we should keep in mind that PCSK9 overexpression has been associated with pro-angiogenic features in vitro, as it may promote the release of VEGF. This is a significant drawback regarding their potential beneficial role in malignancy [161]. In conclusion, while PCSK9 inhibition shows promise as a therapeutic strategy against cancer, its effects are complex and may differ depending on the type of cancer and specific cellular contexts.

### 3.4. Bempedoic Acid

Bempedoic acid is a novel hypolipidemic agent approved by the Food and Drug Administration (FDA) in 2020 for treating dyslipidemia. It acts by inhibiting ACLY, resulting in LDL-C level reductions [162]. As a monotherapy, BA lowers LDL-C by up to 30% and boosts the effects of high-intensity statins by approximately 15%. A daily combination of BA at 180 mg, ezetimibe at 10 mg, and atorvastatin at 20 mg results in an LDL-C decrease of nearly 60% in hypercholesterolemia patients [163]. BA is also an alternative option for individuals who exhibit intolerance to statins, as it does not cause myopathy [164].

#### 3.4.1. Inflammation

Bempedoic acid offers CVD reduction, likely due to both its lipid-lowering and anti-inflammatory properties. The anti-inflammatory features of BA are primarily achieved through the upregulation of AMPK in immune cells, which attenuates the activation of MAPK pro-inflammatory pathways. This results in a reduction in the production of chemokines, cytokines, and adhesion molecules, thereby decreasing leukocyte accumulation and activation in the artery subendothelium and visceral adipose tissues [165].

Experimental evidence from diet-induced obese mice has demonstrated that BA administration can restore AMPK activity in adipose tissue, leading to a reduction in IL-6 production and tissue inflammation [166]. Given that IL-6 promotes the production of CRP by the liver, it is not surprising that individuals receiving BA may exhibit a 20–30% reduction in hsCRP levels [167]. A pooled analysis of four CLEAR randomized trials evaluating BA in subjects with hypercholesterolemia and ASCVD, whether on statin therapy or statin-intolerant, revealed a significant reduction in hs-CRP levels. The decrease was 18.1% in individuals on statin treatment and 27.4% in statin-intolerant patients, indicating a potent anti-inflammatory effect [168]. Furthermore, the BA-induced inhibition of ACLY appears to facilitate a reduction in prostaglandin E2 production, further alleviating the inflammatory response [169].

#### 3.4.2. Vascular Health

Bempedoic acid has been associated with potential endothelial dysfunction because of its ability to cause hyperuricemia. Elevated uric acid levels can contribute to hypertension by increasing systemic vascular resistance and decreasing NO availability [170]. However, recently, it has been reported that bempedoic acid may have unexpected antihypertensive effects and improve vascular health. This potential benefit could be due to its capacity to counteract the harmful effects of chronic activation of the Ang II receptor 1 (AT-1R) on cellular protection and survival. BA activates the adenosine monophosphate-activated AMPK signaling pathway, leading to higher NO production and potential reduction in ER stress. Additionally, bempedoic acid might exhibit anti-fibrotic properties and prevent vascular remodeling by inhibiting the extracellular signal-regulated kinase (ERK)/transforming growth factor-β fibrotic signaling pathway [171]. 

#### 3.4.3. Glucose Metabolism

Bempedoic acid may positively affect glucose metabolism and insulin sensitivity due to its combined impact on ACLY inhibition and AMPK activation. ACLY inhibition reduces glucose synthesis by limiting oxaloacetate availability for gluconeogenesis [12,172,173]. Additionally, AMPK activation regulates glucose production and energy balance by downregulating key enzymes involved in glucose production, such as glucose-6-phosphatase (G6Pase) and phosphoenolpyruvate carboxykinase (PEPCK). G6Pase hydrolyzes glucose-6-phosphate to produce free glucose for release from the cell, while PEPCK catalyzes the initial step of gluconeogenesis, converting oxaloacetate into phosphoenolpyruvate [173,174].

#### 3.4.4. Malignancy

ACLY is often overexpressed in many cancers and plays a central role in cancer metabolism by depleting cytosolic citrate, which enhances glycolysis through increased PFK1 and PFK2 activity and activates oncogenic drivers such as PI3K/AKT. Therefore, inhibiting ACLY with BA may help mitigate tumor development [175]. Recent evidence suggests that BA may be a potential treatment for head and neck squamous cell carcinoma (HNSCC). This may be attributed to its ability to inhibit ACLY, which is abundant in small extracellular vesicles (sEVs) derived from interleukin-8-activated CXCR1High cells. ACLY promotes the transformation of CXCR1Low to CXCR1High cells, aiding HNSCC progression by acetylating NF-κB p65 and facilitating its nuclear translocation to transcribe CXCR1 [176]. 

Furthermore, BA has shown promise for individuals undergoing treatment for breast and pancreatic cancer with palbociclib, a CDK4/6 inhibitor. When palbociclib activates ACLY, combining it with BA inhibits ACLY, reducing tumor cell viability and enhancing apoptosis, thereby impeding cell invasion [177]. Interestingly, BA has been linked to alterations in intracellular isoprenoid composition, which can significantly impact protein prenylation. As such, bempedoic acid may function as a prenyltransferase inhibitor. PTases serve as posttranslational modifiers of proteins involved in various cellular processes, rendering them potential drug targets for a broad spectrum of disorders, including malignancy and AD [178]. 

**Figure 1:**
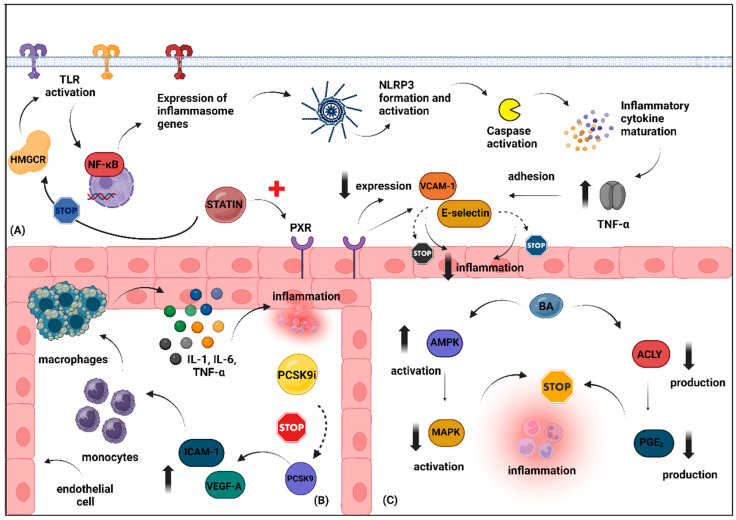
Schematic illustration depicting the potential anti-inflammatory effects of hypolipidemic agents primarily targeting LDL-C reduction. (**A**). Typically, HMGCR facilitates the activation of TLRs, which subsequently triggers the expression of inflammasome-related genes through the NF-kB signaling pathway. This leads to inflammasome formation and the activation of caspase-1 protein, resulting in the maturation of several pro-inflammatory cytokines, including TNF-a. TNF-a promotes the adhesion of various molecules, such as VCAM-1 and E-selectin, contributing to endothelial dysfunction. Statins may exert anti-inflammatory effects either by inhibiting HMGCR or by enhancing the expression of PXR, a receptor commonly found in the vasculature. Activation of PXR reduces the expression of these adhesion molecules, thus promoting vascular detoxification and reducing vascular inflammation. (**B**). PCSK9 increases the expression of VEGF-A and ICAM-1 adhesion molecules, which leads to the migration of monocytes and macrophages, thereby boosting the production of pro-inflammatory cytokines and causing endothelial dysfunction. Silencing PCSK9 may counteract these effects. (**C**). Bempedoic acid exerts its anti-inflammatory effects primarily by upregulating AMPK, which subsequently reduces the activation of MAPK pro-inflammatory pathways. Additionally, bempedoic acid may improve the inflammatory environment by decreasing the production of prostaglandin E2 through the inhibition of ACLY. Abbreviations: ACLY, ATP-citrate lyase; AMPK, AMP-activated protein kinase; BA, bempedoic acid; HMGCR, 3-hydroxy-3-methylglutaryl-CoA reductase; ICAM-1, intercellular adhesion molecule-1; LDL-C, low-density lipoprotein cholesterol; MAPK, mitogen-activated protein kinase; NF-kB, nuclear factor kappa B; PCSK9, proprotein convertase subtilisin/kexin type 9; PCSK9 i, proprotein convertase subtilisin/kexin type 9 inhibitor; PXR, pregnane X receptor; TLR, toll-like receptors; TNF-α, tumor necrosis factor-alpha; VCAM-1, vascular cell adhesion molecule-1; VEGF-A, vascular endothelial growth factor A. Created with www.BioRender.com. (assessed on 14 July 2024).

In conclusion, the landscape of lipid-lowering treatments that primarily reduce LDL-C has been extensively explored, with statins and ezetimibe being the most researched. Statins are known for their multiple beneficial effects, including enhancing vascular function and reducing inflammation, although they have diabetogenic concerns and mixed evidence regarding cancer risk. Ezetimibe also contributes significantly to cardiovascular health beyond cholesterol reduction, showing anti-inflammatory benefits and potential improvements in vascular health, albeit with inconsistent effects on glucose metabolism and cancer outcomes. PCSK9 inhibitors emerge as powerful agents, excelling in LDL-C reduction and exhibiting anti-inflammatory and potential anticancer properties. Last, bempedoic acid is recognized for its effectiveness in managing dyslipidemia, along with promising roles in addressing inflammation, vascular health, glucose metabolism, and malignancy, underscoring its versatility in contemporary therapeutic strategies. Figure 1 illustrates different anti-inflammatory mechanisms through which the aforementioned agents exert their pleiotropic effects, as evidenced by experimental studies.

## 4. Thinking Pleiotropic in Familial Hypercholesterolemia

### 4.1. Mipomersen and Lomitapide

Mipomersen and lomitapide are lipid-lowering agents primarily recommended for individuals with homozygous familial hypercholesterolemia (HoFH). Mipomersen, a second-generation antisense oligonucleotide inhibitor of ApoB100, promotes the selective degradation of ApoB100 mRNA, independently of LDL-R function, thereby reducing the production of ApoB100-enriched lipoproteins, including LDL-C, VLDL-C, and Lp(a) [13]. Lomitapide, on the other hand, is an oral selective inhibitor of MTP, which affects the production of VLDL lipoproteins that primarily contain ApoB100. Comparatively, lomitapide is thought to exhibit more robust lipid-lowering effects, reducing LDL-C by about 50%, whereas mipomersen achieves approximately a 25% reduction in LDL-C levels. Despite their promise in treating FH, the potential side effects of these agents, particularly their propensity to cause hepatic steatosis, have limited their clinical use [13,14]. 

Regarding their pleiotropic effects, data on mipomersen are limited. In contrast, recent experimental studies have explored the potential pleiotropic effects of lomitapide, including its anti-inflammatory and anti-cancer properties. Preliminary evidence suggests that lomitapide administration may enhance vascular function in obese mice by alleviating body weight gain and restoring lipid profiles, with favorable effects on inflammation and oxidative stress, respectively [179]. Additionally, lomitapide has demonstrated anti-cancer properties by facilitating autophagic cell death via mTOR inhibition and by promoting anti-proliferative effects in preclinical pancreatic cancer models [180]. Moreover, lomitapide may offer benefits when combined with immune checkpoint-blocking antibodies, as this combination has been shown to reduce tumor growth in murine preclinical syngeneic tumor models [181]. Lomitapide’s effects may also extend to hematological malignancies, with recent evidence indicating that targeting PARP14 with lomitapide attenuates drug resistance via DRP1-induced mitophagy activation in multiple myeloma [182]. PARP14, overexpressed in multiple myeloma, functions as a transcriptional co-activator for STAT6, facilitating the Th2 immune response, promoting metabolic changes to an anaerobic state, and activating cell survival pathways through JNK2 and the PGI/AMF complex [183].

### 4.2. ANGPTL3 Inhibitors

Recently, the FDA sanctioned a pioneering category of cholesterol-lowering medications termed angiopoietin-like 3 protein (ANGPTL3) inhibitors. These compounds operate by obstructing ANGPTL3, thereby amplifying the functions of LPL and EL. Consequently, LPL and EL aid in the elimination of remnants of VLDL via liver receptors, culminating in a decrease in LDL-C levels irrespective of LDL-R activity. ANGPTL3 inhibitors find their primary application in refractory homozygous FH [184]. Presently, evinacumab and vupanorsen stand as the principal ANGPTL3 inhibitors in clinical application, furnishing LDL-C reductions spanning 18–25% [185,186]. Recent studies have illuminated the potential positive impacts of ANGPTL3 inhibition extending beyond lipid metabolism, underscoring their prospective influence, particularly on inflammation and malignancy.

#### 4.2.1. Inflammation

Evidence from experiments with cultured THP-1-derived macrophages reveals that human recombinant ANGPTL3 elevates the levels of proinflammatory cytokines IL-1β, IL-6, and TNF-α, underscoring the direct involvement of ANGPTL3 in inflammation. Both in vivo and in vitro data have indicated that knocking out Angptl3 may provide advantages in instances of renal dysfunction. This is achieved by promoting the conversion of M1 macrophages to M2 macrophages and reducing the activation of the NLRP3 inflammasome [187]. Beyond its secreted form, the intracellular overexpression of ANGPTL3 also significantly impacts inflammatory responses. Recently, Zhang et al. have shown that elevated ANGPTL3 levels inhibit IL-1β-induced NF-κB activation and the transcription of inflammatory genes in the liver, whereas this inhibitory effect is reversed when ANGPTL3 expression is knocked down. ANGPTL3 accomplishes this by interacting with IL1R1 and IL1RAP through its intracellular C-terminal FLD, thereby disrupting the assembly of the IL1R1-associated complex, which is critical for IL-1β-induced signaling. These insights highlight a new role for ANGPTL3 in inflammation, where it interferes with the normal interaction between IL1R1 and IL1RAP, thereby maintaining immune tolerance and homeostasis in the liver [188]. 

#### 4.2.2. Vascular Health

ANGPTL3 plays a significant role in inflammation by modulating macrophage activity within atheromatous lesions through its C-terminal fibrinogen-like domain (FLD). Unlike FLDs from other angiopoietins that bind to endothelial cells via Tie receptors, the ANGPTL3 FLD specifically targets integrin αVβ3, a key component in the formation of atherosclerotic plaques [189]. This binding promotes foam cell formation by decreasing the expression of scavenger receptor A (SRA) and CD36 while simultaneously enhancing pro-angiogenic activities. Furthermore, the interaction of ANGPTL3 with αvβ3 integrin in endothelial cells facilitates endothelial cell adhesion and migration. This interaction also activates intracellular signaling pathways, such as mitogen-activated protein kinase and Akt phosphorylation, which can lead to direct vascular injury [190]. In patients undergoing maintenance hemodialysis, where endothelial dysfunction is prevalent, ANGPTL3 levels have been found to negatively correlate with the vascular reactivity index (VRI), an indicator of endothelial function [191]. 

#### 4.2.3. Glucose Metabolism

While the role of ANGPTL3 in lipid metabolism is well-established, its impact on glucose metabolism remains unclear. Preliminary evidence using CRISPR-Cas9 to create ANGPTL3 knockout mice suggests that ANGPTL3 loss-of-function (LOF) may improve insulin sensitivity. This improvement might be due to increased LPL activity, which benefits insulin sensitivity and lowers free fatty acid levels in the bloodstream. This reduction in free FAs may result from impaired mobilization from adipose tissue [192]. Conversely, ANGPTL3 has been shown to enhance endogenous adipogenesis, which can reduce insulin sensitivity [193]. Thus, ANGPTL3 inhibition may potentially offer benefits related to glucose metabolism in individuals with T2DM.

#### 4.2.4. Malignancy

Recent evidence has underscored the involvement of ANGPTL3 in the occurrence, development, and survival rates of various malignancies. Particularly, ANGPTL3 has been found to be elevated in gastrointestinal cancers, including esophageal and hepatocellular carcinoma [194,195]. Moreover, recent data highlight its involvement in colorectal cancer (CRC), as individuals with CRC exhibit upregulated ANGPTL3 expression, leading to worse survival rates. ANGPTL3 overexpression appears to facilitate the proliferation and migration of CRC cells partially via mitogen-activated protein kinase 14 (MAPK14). Interestingly, these findings may be reversed by ANGPTL3 inhibition, while ANGPTL3 downregulation may suppress tumor growth and liver metastasis. Therefore, ANGPTL3 inhibition may represent a novel treatment strategy for individuals with CRC [196].

On the other hand, data regarding the role of ANGPTL3 in ovarian cancer are conflicting. Some data suggest that increased ANGPTL3 expression is associated with shorter survival in individuals with high-grade serous ovarian cancer (HGSC) [197]. However, recently, Wu et al. have shown that ANGPTL3 inhibition may have a negative impact on individuals with ovarian cancer, as ANGPTL3 is reduced in ovarian cancer tissues and cells, predicting a favorable prognosis. Specifically, an increase in ANGPTL3 mitigates the proliferation of ovarian cancer cells in vitro and the metastatic potential of these cells. Furthermore, ANGPTL3 promotes natural killer (NK) cell killing of ovarian cancer cells while also modulating the JAK/STAT3 pathway to influence metastasis and immune resistance [198].

### 4.3. Lipoprotein Apheresis

Lipoprotein apheresis is a therapeutic modality designed to selectively eliminate apoB-containing lipoproteins from circulation [199]. While LA has been shown to reduce LDL-C and Lp(a) concentrations significantly, its clinical impact on CVD risk mitigation is still conflicting [200]. LA is typically recommended for individuals with FH, particularly those with the homozygous form, who fail to achieve therapeutic goals despite optimal tolerated lipid-lowering treatments [201]. Particularly in Germany, LA is indicated for patients with progressive CVD and Lp(a) concentrations exceeding 60 mg/dL, regardless of LDL-C levels [202]. Several methods are available for performing LA, including adsorption, precipitation, and filtration techniques [203]. Beyond its central role in reducing lipoprotein levels, LA has been found to offer various pleiotropic benefits that may contribute to its therapeutic effects. These benefits include reductions in inflammation and enhancements in vascular health, including improvements in endothelial function and blood viscosity [204]. 

Research indicates that in individuals with severe hypercholesterolemia, lipoprotein apheresis may possess anti-inflammatory effects, interfering with different pathways of the inflammatory process. LA appears to influence the complement system differently depending on the LDL apheresis system used. Plasma separation columns tend to promote the formation of proinflammatory complement factors C3a and C5a, whereas LDL apheresis columns adsorb these anaphylatoxins to different extents [205]. LA may also mitigate inflammation by reducing the mRNA expression of pro-inflammatory molecules such as IL-1α, IL-6, and TNF-α [8]. Additionally, LA has shown benefits in reducing CVD-related inflammatory biomarkers such as CRP and fibrinogen, as evidenced by several studies [206,207,208]. 

However, evidence regarding the effect of LA on myeloperoxidase (MPO) levels in hypercholesterolemic patients is conflicting. Some studies have reported an increase in MPO post-LDL apheresis, while others have observed a reduction, often linked to changes in total cholesterol levels [209]. MPO is a heme-binding protein associated with proinflammatory properties implicated in atherosclerotic plaque development, though it’s also suggested to play a role in ending the inflammatory process [210]. Notably, while variations exist among LDL apheresis columns and patient demographics across studies, most agree that pro-inflammatory cytokines are partially removed by the LDL apheresis columns, with some studies noting an increase in anti-inflammatory cytokines during the procedure [211,212]. 

Additionally, LA might affect the immune system, potentially influencing certain T cell and NK cell subsets expressing CD69 [213]. Multiple LA sessions have been linked to a decrease in these CD69-expressing cell subsets. T cells are pivotal in early-stage atherosclerosis and are prevalent in advanced atheromatous plaques [214]. Conversely, CD69, an immune activation marker, plays a multifaceted role in inflammation; its presence can promote inflammation, whereas its absence is associated with susceptibility to inflammatory or autoimmune disorders. This hints at a CD69 immunoregulatory function, potentially amplifying the suppressive role of regulatory T cells (Tregs). Hence, the modulation of CD69 expression by LA may contribute to immune regulation, impacting inflammatory processes such as atherosclerosis [215]. 

The oxidative modification of LDL-C is a crucial factor in the early stages of atherosclerosis, and lipid peroxidation is notably higher in patients with FH. Lipoprotein apheresis has been shown to reduce oxidative stress, suggesting that reductions in lipid peroxidation parallel to decreases in LDL-C concentrations make LA an effective method for preventing cardiovascular disease in FH patients [216]. In a recent study involving eleven patients with FH and hyperlipoproteinemia(a) treated with regular LA using the direct adsorption of lipoproteins(DALI) or membrane filtration optimized novel extracorporeal treatment (MONET) techniques, a single LA session resulted in similar reductions in lipid-related oxidative stress markers. Specifically, concentrations of 8-iso-prostaglandin F2α (8-isoPGF2) and thiobarbituric acid reactive substances (TBARS) were reduced by approximately 60% and 30%, respectively [217]. Similarly, a clinical study conducted in China with 31 FH patients reported significant reductions in free oxygen radicals test (FORT) values and significant increases in free oxygen radicals defense (FORD) values immediately after LA treatment compared with pre-treatment levels [218]. 

Years ago, evidence indicated that repeated LA may lead to hemodynamic alterations in individuals with FH [219]. Although most studies involve a small number of patients, likely due to the rare indication of LA use, a considerable number of studies have reported that LA may reduce blood viscosity, typically assessed using the Rheolog capillary viscometer [220]. Importantly, this reduction persists even one week after treatment [221]. 

The aforementioned beneficial effects of repeated LA have been demonstrated in a 6-month follow-up period, wherein LA resulted in improvements in multiple CVD-related parameters, including circulating endothelial progenitor and mature cells, flow-mediated vasodilatation, left ventricular ejection fraction, homocysteine levels, and microalbuminuria [199]. Interestingly, recent data demonstrate that in subjects with increased serum Lp(a) levels, LA may also eliminate extracellular vesicles (EVs) [222]. EVs are cell-derived particles that mediate intercellular communication by transporting various molecules, including proteins, lipids, and cytokines [223]. These particles are highly involved in maintaining homeostasis, inflammation, neo-angiogenesis, and thrombosis, all of which are pivotal underlying substrates of ASCVD [224]. Thus, EVs may serve as potential CVD biomarkers, useful for assessing the severity and progression of ASCVD [225]. 

Overall, therapies tailored for individuals with FH demonstrate a variety of pleiotropic effects extending beyond their primary mechanisms. Lomitapide, for instance, exhibits anti-inflammatory and anti-cancer properties, while ANGPTL3 inhibitors offer versatility by impacting inflammation, vascular health, glucose metabolism, and malignancy. Lipoprotein apheresis similarly provides significant pleiotropic benefits, primarily through its anti-inflammatory actions and improvements in vascular health. Understanding and leveraging these diverse effects is critical for optimizing therapeutic outcomes and broadening the clinical applications of these treatments beyond lipid modulation.

## 5. The Pleiotropic Effects of HDL-C Enhancement: Exploring Old and Novel Agents

### 5.1. Niacin

Niacin, also known as vitamin B3, comprises nicotinic acid and nicotinamide, which play pivotal roles in numerous biochemical reactions within the human body [226]. When administered at doses of 1–2 g/day, niacin can modestly improve HDL-C levels, typically increasing HDL-C by up to 20–25% while also reducing TGs and LDL-C [227]. Despite these beneficial effects on lipid profiles, niacin use has not been linked to significant reductions in total or cause-specific mortality or recurrent cardiovascular events in individuals with or at risk of ASCVD [228]. Nevertheless, several studies have underscored niacin’s potential anti-inflammatory properties, improvements in vascular health, and possible anti-cancer effects.

#### 5.1.1. Inflammation

Niacin possesses both direct and indirect anti-inflammatory properties, primarily through the activation of the hydroxycarboxylic acid receptor 2 (HCA2) receptor in various tissues. It inhibits the expression of adhesive molecules in blood vessels and suppresses pro-inflammatory cytokines in adipose tissue. In monocytes, it reduces the secretion of inflammatory markers such as TNF-α and IL-6. Research involving animals and human cells demonstrates that niacin can mitigate inflammation by downregulating the NF-κB pathway and reducing the expression of inflammatory molecules. These effects occur independently of changes in lipid levels [229]. Additionally, niacin exhibits anti-oxidative properties, which may be due to its ability to enhance HDL-C levels or activate Nrf2, a transcription factor crucial for regulating mechanisms affecting oxidative stress and subsequent inflammation [230,231].

The anti-inflammatory benefits of niacin have also been demonstrated in experimental settings of CKD, where its administration has been shown to reduce oxidative stress and inflammation. Notably, niacin alleviates MCP-1, PAI-1, TGF-β, and NF-κB activation, leading to improvements in hypertension, proteinuria, glomerulosclerosis, and tubulointerstitial injury [232]. A recent meta-analysis by Rad et al. highlighted that niacin usage is associated with significant reductions in CRP and TNF-α levels, further supporting its potential anti-inflammatory effects. Moreover, niacin positively affects adipokines, increasing levels of adiponectin and leptin, thereby debilitating metabolic dysregulation [233].

#### 5.1.2. Vascular Health

Years ago, niacin was reported to enhance endothelial function, likely due to its ability to raise HDL-C levels. The INEF study, a large-scale randomized, double-blind, placebo-controlled trial, found that extended-release niacin at 1000 mg daily might reduce endothelial dysfunction in subjects with CAD and low HDL-C but not in those with normal HDL-C [234]. Subsequent studies have explored the beneficial impact of niacin on endothelial function. Kaplon et al. demonstrated that higher dietary niacin intake is linked to enhanced vascular endothelial function and reduced systemic and vascular oxidative stress among healthy middle-aged and older subjects [235]. Recent research further confirms niacin’s potential in enhancing endothelial function and reducing vascular aging, showing that niacin activates Sirt1, a key regulator of vascular endothelial NO production [236]. 

Moreover, niacin at low concentrations (10 μM) appears to enhance human microvascular endothelial cell (HMVEC) angiogenic function under lipotoxic and hypoxic conditions. This effect is likely independent of lipid correction and is mediated via the activation of the niacin receptor GPR109A, which is expressed in endothelial cells [237]. Additionally, niacin promotes revascularization and functional recovery of the hind limb following ischemic injury in diet-induced obese mice with hyperlipidemia, highlighting its potential in treating peripheral ischemic vascular disease associated with metabolic syndrome [238]. 

Notably, niacin has shown promise in cases of ischemic stroke, potentially reducing thrombotic risk. The main mechanism proposed is niacin’s ability to reduce platelet aggregation and support blood clot breakdown. Niacin also decreases TGF-β-induced increases in PAI-1 and fibrinogen levels [239]. The positive effects of niacin on endothelial function were confirmed in a meta-analysis by Sahebkar. This analysis included seven RCTs with a total of 441 participants and concluded that niacin supplementation may improve endothelial function, indicated by a weighted mean increase in FMD of 1.98% [240]. Nevertheless, despite the plethora of evidence supporting niacin’s favorable role in vascular health, recent findings from Ferrell et al. contradict these effects. They have shown that the terminal breakdown products of excess niacin, 2PY and 4PY facilitate vascular inflammation, an association that may translate to the presence of residual CVD risk [241].

#### 5.1.3. Glucose Metabolism

Niacin is known for its tendency to promote mild increases in blood glucose levels in both diabetic and non-diabetic individuals, highlighting the importance of monitoring glucose levels during its administration. Among 17 non-diabetic postmenopausal women, niacin has been reported to significantly impact mean glucose, insulin, and C-peptide levels, leading to increases of 10.6%, 61.8%, and 46.1%, respectively [242]. Additionally, long-term treatment with niacin has been proposed to negatively impact glucose homeostasis, although the underlying mechanisms remain unclear. Niacin’s hyperglycemic effect is presumably associated with its ability to impair pancreatic islet function by reducing glucose-stimulated insulin secretion partially through the upregulation of GPR109A and PPARγ2 [243,244]. Nevertheless, experimental evidence supports the antidiabetic potential of niacin supplementation in diabetic rats, demonstrating that its administration re-results in hyperglycemia control in a dose-dependent manner, with notable reductions in fasting glucose levels. Niacin is also suggested to alleviate oxidative stress, which is essential in the onset and progression of T2DM, further diminishing DNA damage and tissue injury caused by ROS production [245].

#### 5.1.4. Malignancy

Mounting evidence highlights niacin’s potential beneficial impact on malignancy, including reduced mortality rates, as demonstrated by the NHANES retrospective cohort study involving 3504 individuals [246]. Niacin exerts a significant influence on DNA repair and genomic stability, crucially affecting cancer risk through its derivative nicotinamide adenine dinucleotide (NAD^+^). NAD is pivotal in ADP-ribosylation processes, including the activity of poly(ADP-ribose) polymerase-1 (PARP-1), essential for DNA damage response, repair, and stress signaling. Moreover, NAD^+^ plays a critical role in calcium signaling pathways; disruptions can lead to genomic instability and heightened cancer susceptibility, evident in both solid tumors and hematological malignancies such as skin cancer and leukemia [247]. 

In combating colon cancer, niacin has shown promise by activating autophagic flux in human colon cancer cells, protecting them from mitochondrial dysfunction and TRAIL-induced apoptosis [248]. Additionally, recent data illuminate niacin’s anti-cancer properties and its potential role in mitigating chemotherapy side effects. Notably, niacin appears effective in ameliorating cancer-related cachexia, a condition exacerbated by both cancer and chemotherapy. Experimental and clinical evidence reveals that severe cachexia correlates with depleted NAD^+^ levels and reduced Nrk2 activity, an enzyme involved in NAD^+^ biosynthesis. Niacin supplementation restores tissue NAD^+^ levels, enhances mitochondrial metabolism, and alleviates cachexia induced by cancer and chemotherapy [249]. However, certain malignancies, particularly those exhibiting BRCAness, exploit niacin by upregulating NAPRT, accelerating niacin conversion to NAD^+^, and enhancing DNA repair, thereby promoting tumor progression [250]. Additionally, Tosti et al. suggest that restricting niacin, in combination with inhibiting NAMPT, an enzyme in niacin metabolism, leads to synthetic lethality in neuroendocrine carcinomas of the lung and prostate [251]. Furthermore, data from a meta-analysis in both immunocompetent and immunosuppressed patients indicate insufficient evidence that niacin treatment significantly diminishes keratinocyte cancers [252].

### 5.2. CETP Inhibitors

Cholesteryl ester transfer protein (CETP) facilitates the movement of cholesteryl esters from HDL-C particles to lipoproteins containing ApoB. Blocking CETP leads to a marked rise in HDL-C levels and a simultaneous decrease in both LDL-C and Lp(a) levels [253]. Although CETP inhibitors have shown impressive lipid-altering capabilities, they have not yielded notable cardiovascular benefits. Nevertheless, novel research indicates that CETP inhibitors may positively impact various other pathological conditions, highlighting their potential advantages beyond reducing CVD risk [254].

#### 5.2.1. Inflammation

Given that CETP inhibitors promote increases in HDL-C and apolipoprotein A1 (ApoA1) levels, it is reasonable to consider that these agents might offer anti-inflammatory benefits, as both HDL-C and ApoA1 are known for their anti-inflammatory and antioxidant properties. HDL-C has been shown to reduce foam cell formation, prevent LDL-C oxidation, and decrease tissue neutrophil infiltration [255,256]. Similarly, ApoA1 can attenuate the activation of macrophages by T-cells, thereby reducing the production of inflammatory cytokines and chemokines [257]. However, some data indicate that CETP inhibitors might actually enhance inflammation by increasing serum levels of hsCRP [258]. Additionally, early evidence suggests that despite improvements in HDL-C functionality, CETP inhibition may impair the anti-inflammatory capacity of HDL-C, potentially leading to fatty liver and insulin resistance, particularly in the context of obesity [259].

Conversely, data from Mendelian randomization studies have shown a causal relationship between HDL-C and IBD, indicating that genetically proxied CETP inhibition may reduce the risk of Crohn’s disease [260]. Emerging research also underscores the potential of CETP inhibitors in treating various conditions where inflammation is critical, including neurodegenerative diseases such as Alzheimer’s disease, age-related macular degeneration (AMD), and severe inflammatory states such as sepsis [254].

CETP inhibitors may hold significant promise for AD therapy. Preclinical studies and epidemiological evidence suggest that higher HDL-C levels are associated with reduced AD risk, implying that CETP inhibition could represent a novel treatment option for AD. These inhibitors reduce overall brain cholesterol content and apolipoprotein E4 (ApoE4) concentration while elevating apoA1/pre-beta HDL levels in the choroid plexus and bloodstream, thus improving cholesterol metabolism in the neurovascular unit [261]. Individuals with loss-of-function mutations in the CETP gene are shielded from the AD risk associated with carrying an ApoE4 mutation [262]. Conversely, AD patients harboring an ApoE4 allele typically exhibit lower HDL-C and ApoA1 levels [263]. This holds significance as apoA1 overexpression mitigates neuroinflammation, safeguards against cerebral amyloid angiopathy, and helps prevent cognitive dysfunction [264,265].

Enhancing HDL-C through CETP inhibition may also increase the bioavailability of xanthophylls, potentially offering protection against AMD. AMD develops when the macular surface, rich in photoreceptors, is compromised by the accumulation of lipoprotein-rich deposits known as drusen, and inflammation is highly involved in AMD pathogenesis [266]. Both preclinical and clinical findings suggest that delivering lipophilic antioxidants to the macula can provide protective benefits against AMD [267,268]. Since HDL-C particles are the primary carriers of xanthophylls, it is hypothesized that boosting HDL-C levels could improve the circulation and availability of these beneficial antioxidants [269]. By enhancing the transport of xanthophylls, which include lutein and zeaxanthin, HDL-C may help mitigate oxidative stress and inflammation in the macula, thereby reducing the risk or progression of AMD [270,271].

Mounting evidence suggests that inhibiting CETP may also be beneficial in severe inflammatory states, such as sepsis. Sepsis often leads to reduced levels of HDL-C and its associated apolipoproteins, ApoA1, and apolipoprotein C1 (ApoC1) [272,273]. Preclinical evidence has shown that administering HDL-C or genetically increasing apoA1 expression can alleviate inflammation and improve survival rates in intra-abdominal sepsis. For instance, in mice with polymicrobial sepsis, CETP inhibition helped maintain plasma HDL-C and apoA1 levels, resulting in higher survival rates compared with the placebo [274]. Genetic data have corroborated these findings, as a gain-of-function variant, rs1800777-A, has been linked to a significant decline in HDL-C levels during sepsis, increasing the risk of organ failure and mortality [275].

Regarding the underlying mechanism, it is hypothesized that HDL-related apolipoproteins can bind to and facilitate the removal of lipopolysaccharide (LPS), a component of Gram-negative bacteria, from the bloodstream. In cases of Gram-negative-induced sepsis, the release of LPS endotoxin activates Kupffer cells, which exert antibacterial effects by releasing pro-inflammatory cytokines and reducing CETP expression, thereby increasing HDL-C levels in the bloodstream. Consequently, HDL-C helps neutralize systemic endotoxemia by binding to LPS and promoting a proinflammatory reaction in macrophages, aiding in bacterial clearance [276,277].

#### 5.2.2. Vascular Health

The effects of CETP inhibitors on endothelial function are still debated. Recent findings suggest that CETP presence in endothelial cells contributes to vascular oxidative stress and endothelial dysfunction. In cultured human aortic endothelial cells, CETP inhibition reduced oxidative stress from major ROS sources, including mitochondria and NO_X_2. Additionally, markers of endoplasmic reticulum stress and inflammatory molecules involved in atherosclerosis, such as TNF-α, ICAM-1, and VCAM-1, were alleviated [278]. However, experimental data show mixed results for specific CETP inhibitors. For instance, evacetrapib did not improve endothelial function, and anacetrapib might have adverse effects on the endothelium [279].

The influence of CETP on endothelial function also appears to be sex-dependent. In male mice, expression of the human CETP gene impaired endothelium-mediated vascular relaxation, correlating with oxidative stress. In contrast, increased CETP expression in female mice improved endothelial function via estrogen receptor-α and the endothelial nitric oxide synthase pathway, leading to reduced ROS production and increased eNOS-derived NO, which promotes anticontractile effects [280]. These varying effects of CETP inhibition on vascular function may partly explain the failure of CETP inhibitors to reduce cardiovascular risk despite significant increases in HDL-C levels observed after their administration.

#### 5.2.3. Glucose Metabolism

CETP inhibition has been shown to reduce the risk of new-onset T2DM in major clinical trials, primarily due to its ability to increase HDL-C, which has glucose-lowering properties. In pancreatic islet β-cells, HDL-C promotes euglycemia by reducing β-cell apoptosis via ATP binding cassette transporters, ABCA1 and ABCG1, and by enhancing insulin secretion through apoA1 and ABCA1/ABCG1 pathways [281]. Additionally, high HDL-C levels improve muscle insulin sensitivity and stimulate insulin-dependent glucose uptake via AMPK and ApoA1 [282]. Preliminary data suggest that specific CETP inhibitors such as anacetrapib, dalcetrapib, and torcetrapib may promote insulin secretion and β-cell survival by stimulating reverse cholesterol transport in β-cells [283,284].

The beneficial effects of HDL-C are diminished when HDL-C levels are low or its metabolic actions are dysregulated, leading to glucose metabolism disruptions and elevated serum glucose levels. Genetic studies on the role of CETP in T2DM prevention have yielded conflicting results [285,286]. However, a recent larger study indicated that CETP inhibition could be beneficial for preventing T2DM by analyzing genetic associations with LDL-C, HDL-C, and TG as proxies for lower CETP concentration or activity [287].

#### 5.2.4. Malignancy

**Figure 2:**
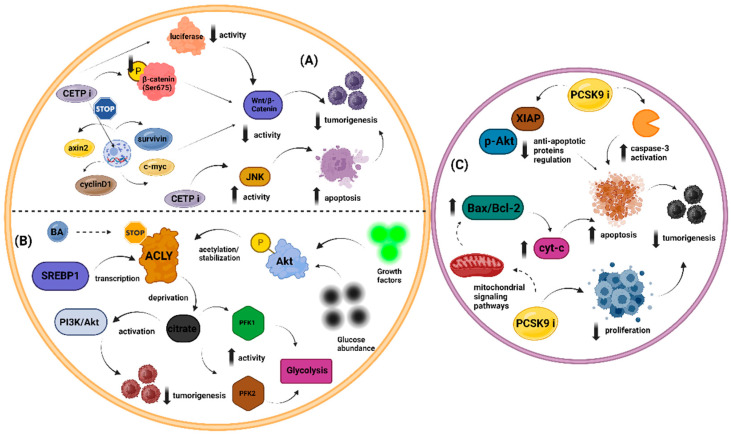
Emerging evidence regarding the anti-cancer features of novel lipid-lowering treatments, including CETP inhibitors, bempedoic acid, and PCSK9 inhibitors. (**A**). CETP inhibitors may confer anti-cancer effects by inhibiting the Wnt/β-Catenin signaling pathway and activating the JNK signaling pathway. The activation of JNK by CETP inhibition exhibits apoptotic activity, resulting in diminished tumorigenesis. Additionally, CETP inhibition may reduce tumorigenesis by dampening the activation of Wnt/β-Catenin. Specifically, CETP silencing has been associated with decreased activation of luciferase, reduced levels of the phosphorylated form of β-catenin (p-β-catenin (Ser675)), and lower levels of target genes involved in Wnt signaling, including axin2, cyclin D1, c-myc, and survivin. (**B**). Bempedoic acid has been suggested to possess anti-malignant properties through its ability to inhibit ACLY. SREBP1 promotes ACLY transcription, while the phosphorylation of Akt—mediated by both growth factors and glucose abundance—facilitates the enzyme’s acetylation and stabilization. ACLY activation leads to citrate deprivation, resulting in reduced tumorigenesis by enhancing the activation of the PI3K/Akt pathway. Interestingly, citrate deprivation is also linked to increased activity of regulatory enzymes in glycolysis, specifically PFK1 and PFK2. (**C**). PCSK9 silencing may exhibit anti-cancer properties by either reducing tumor cell proliferation or enhancing cancer cell apoptosis. PCSK9 inhibitors can facilitate apoptosis by increasing the activation of caspase-3 and downregulating anti-apoptotic proteins such as p-Akt and XIAP. Furthermore, PCSK9 inhibition may promote tumor cell apoptosis by interfering with mitochondrial signaling pathways. This interference is associated with an increased Bax/Bcl-2 ratio, leading to the elevated release of cytochrome-c. Abbreviations: ACLY, ATP citrate lyase; Akt, protein kinase B; BA, bempedoic acid; Bax, Bcl-2-associated X-protein; Bcl-2, B cell lymphoma-2; CETPi, cholesteryl ester transfer protein inhibitors; c-myc, cellular myc transcription factor; cyt-c, cytochrome-c; JNK, Jun N-terminal kinase; p-Akt, phosphorylated-Akt; PCSK9 i, proprotein convertase subtilisin/kexin type 9 inhibitor; PFK, phosphofructokinase; PI3K, phosphatidylinositol-3 kinase; SREBP1, sterol regulatory element-binding protein-1; Wnt, wingless-type MMTV integration site family; XIAP, X-linked inhibitor of apoptosis protein. Created with www.BioRender.com. (assessed on 14 July 2024).

About eight years ago, CETP was identified as a potential molecular target for inducing cell death in estrogen-positive breast cancer cells. Esau et al. have demonstrated that MCF-7 CETP knockout breast cancer cells were less resistant to cytotoxic compounds such as tamoxifen and more prone to intrinsic apoptosis [288]. Since then, numerous experimental studies have expanded on this topic. Recently, Gu et al. confirmed the role of CETP in breast cancer growth and aggressiveness, suggesting that CETP inhibition might reduce drug resistance by decreasing cholesterol accumulation in breast cancer cells [289]. Similar benefits have been reported for other types of cancers with the use of specific CETP inhibitors. For instance, Evacetrapib has shown favorable effects against colorectal cancer by inhibiting the Wnt/β-Catenin Signaling Pathway and activating the JNK Signaling Pathway [290]. Table 1 presents both preliminary and clinical studies that have highlighted the pleiotropic benefits of CETP inhibition beyond CVD risk assessment. Subsequently, Figure 2 depicts proposed anti-cancer mechanisms of action of CETP inhibitors, PCSK9 inhibitors, and bempedoic acid, as reported in experimental studies in recent years.

### 5.3. Recombinant HDL-C Particles

Recombinant high-density lipoprotein (rHDL) represents a novel therapeutic approach designed to emulate the beneficial properties of natural HDL-C. Utilizing synthetic or modified apolipoproteins and phospholipids, rHDL effectively promotes reverse cholesterol transport, enhances lipid metabolism, and mitigates atherosclerosis. This innovative technology shows promise in treating cardiovascular diseases by targeting cholesterol efflux pathways. Moreover, rHDL has demonstrated compelling pleiotropic effects, including anti-inflammatory and potential anti-cancer properties [291].

#### 5.3.1. Inflammation

Recent research has increasingly focused on exploring the potential anti-inflammatory benefits of HDL-C, particularly through engineered HDL analogs such as CER001. This synthetic HDL-C particle has shown significant promise in both animal models and clinical settings. In pigs, CER001 demonstrated enhanced median survival rates and substantial reductions in inflammation, complement activation, and endothelial impairment. These findings were further corroborated in humans, where administration of CER-001 led to improvements in ApoA1 levels, enhanced clearance of LPS, and modulation of immune responses. Notably, patients treated with CER-001 exhibited a decreased incidence of severe acute kidney injury (AKI), necessitating less organ support and shorter stays in the intensive care unit [292]. Similarly, the infusion of rHDL particles has shown promise in severe SARS-CoV-2 infections, which often deplete HDL-cholesterol levels and impair HDL-C function. Following rHDL administration, plasma levels of ApoA1 increased while HDL-C levels decreased, indicating efficient cholesterol transport. Proteomic analysis revealed elevated levels of ApoA1 and demonstrated a favorable impact on inflammation, with reductions observed in pro-inflammatory proteins, markers of inflammation, and cytokines [293]. Furthermore, despite not meeting its primary endpoint in the AEGIS-II trial, CSL-112, a formulation of human ApoA1, has shown potential atheroprotective effects through its observed anti-inflammatory properties in animal models and ex vivo studies [294,295].

#### 5.3.2. Vascular Health

Research indicates that rHDL can significantly ameliorate endothelial function through various mechanisms. For instance, reconstituted HDL has been found to restore endothelial function in hypercholesterolemic men by enhancing eNOS activity and increasing NO production. Additionally, these HDL-C particles support endothelial repair by promoting the mobilization and functionality of endothelial progenitor cells, which is crucial for maintaining the integrity of the endothelial layer [296]. rHDL particles also exhibit antioxidative properties, reducing the oxidative modification of LDL and thereby protecting endothelial cells from oxidative stress [297]. Notably, rHDL particles may also positively impact thrombosis as they have been shown to inhibit microvascular and arterial thrombosis by preventing the self-association of von Willebrand factor (VWF), which is crucial for platelet adhesion and aggregation under high shear stress conditions [298].

#### 5.3.3. Glucose Metabolism

rHDL particles have demonstrated potential in mitigating insulin resistance by influencing glucose metabolism through various mechanisms. These include enhancing insulin sensitivity and promoting glucose uptake in tissues independently of insulin. Research has shown that rHDL infusion improves insulin sensitivity in skeletal muscle, a key tissue affected by insulin resistance. Furthermore, HDL particles play a protective role for pancreatic beta cells, shielding them from stress and apoptosis, thereby preserving their function and insulin secretion [299].

#### 5.3.4. Malignancy

In recent years, rHDL particles have gained attention in cancer therapy because of their unique properties and mechanisms that can be harnessed for drug delivery and other therapeutic effects. One notable approach involves synthetic HDL-like nanoparticles (NPs), which show potential for targeting cancer cells and delivering various therapeutic substances. These nanoparticles can be customized to transport small molecules, photothermal agents, and nucleic acids. Additionally, they can function independently as therapeutic agents and deliver imaging substances [300]. Interestingly, rHDL nanoparticles have the unique ability to encapsulate hydrophobic drugs within their core, significantly enhancing the solubility and stability of these drugs in the bloodstream. This targeted delivery mechanism is facilitated through the binding of ApoA1 to scavenger receptor class B type 1 (SR-B1) receptors on cells [301,302]. Tumors frequently exhibit elevated expression of SR-B1 to meet the increased cholesterol demands required for rapid cell proliferation. By exploiting this overexpression, rHDL nanoparticles can efficiently deliver anti-cancer drugs directly to cancer cells, thereby maximizing therapeutic efficacy [302]. Notably, HDL-mediated cholesterol efflux from cancer cells has been shown to inhibit the growth of pancreatic ductal adenocarcinoma (PDAC) cells and induce apoptosis, highlighting the potential of rHDL-based therapies in oncology [303].

In conclusion, while enhancing HDL-C levels has thus far yielded disappointing results in mitigating CVD risk, both niacin and novel agents such as CETP inhibitors and rHDL particles may still hold promise for improving other aspects of human health, such as inflammatory conditions, metabolic syndrome, and cancer. Further research is necessary to fully elucidate these potential benefits, as well as to assess the cost-effectiveness of administering these drugs.

## 6. Pleiotropic Benefits of Pharmaceutical Management of Hypertriglyceridemia

### 6.1. Fibrates

Fibrates are a class of amphipathic carboxylic acids primarily indicated for treating severe hypertriglyceridemia. Their main mechanism of action involves activating the PPAR-α. This activation leads to a substantial reduction in serum triglyceride levels by approximately 50%, a moderate reduction in LDL-C by up to 25%, and an increase in HDL-C by 5–15% [304]. Fenofibrate is the most commonly used fibrate, while bezafibrate and pemafibrate are less frequently prescribed. Beyond their lipid-modifying effects, fibrates exhibit notable pleiotropic properties, with anti-inflammatory features being particularly prominent. These characteristics contribute to their wide-ranging therapeutic benefits across various health conditions.

#### 6.1.1. Inflammation

Fibrates are well-known for their anti-inflammatory properties, which are largely attributed to their lipid-lowering effects, regulation of intracellular lipid metabolism via PPAR-α activation [305], and modulation of specific intracellular signaling pathways [306]. These drugs exert indirect anti-inflammatory effects primarily through their impact on lipid metabolism, which includes lowering LDL-C and increasing HDL-C. This lipid modulation helps reduce cholesterol accumulation in tissues and slows the progression of atherosclerosis, thereby providing anti-inflammatory benefits [307]. Beyond these indirect effects, fibrates also demonstrate direct anti-inflammatory actions independent of their lipid-lowering capabilities [23]. The activation of PPAR-α plays a crucial role in this process by suppressing various inflammation-related intracellular pathways and downregulating genes associated with inflammation [308]. Additionally, fibrates improve mitochondrial function and promote the differentiation of T helper 17 (Th17) cells, contributing further to their anti-inflammatory features [309].

Fenofibrate is particularly noted for its anti-inflammatory potential. It has shown protective effects against osteoarthritis (OA) by preventing senescence, defective autophagy, and inflammation in human OA and aging primary chondrocytes [310]. These findings suggest that fenofibrate may support joint health and cartilage maintenance. However, the presence of diabetes mellitus can alter these effects. Studies on diabetic mice have revealed that fenofibrate may impair bone quality by reducing osteoblast activity, which leads to decreased expression of collagen I and osteocalcin due to downregulated Runx2 expression, a critical factor in bone formation [311].

Similarly, bezafibrate has also been reported to possess anti-inflammatory features in both acute and subacute inflammatory experimental models. Pemafibrate, another fibrate, has shown promise in the treatment of non-alcoholic steatohepatitis (NASH). Evidence suggests that pemafibrate can reduce the expression of the cell adhesion molecule VCAM-1 and favorably modulate inflammation- and fibrosis-related genes in a mouse model of NASH (STAM), highlighting its potential protective role against inflammation and fibrosis in this context [312].

#### 6.1.2. Vascular Health

The impact of hypertriglyceridemia on endothelial function remains a subject of ongoing discussion [313]. While numerous investigations have established a correlation between elevated serum triglyceride levels and endothelial dysfunction, particularly in individuals with conditions such as coronary artery disease and metabolic syndrome [314,315], the precise relationship continues to be debated. Fenofibrate is believed to have benefits for endothelial function primarily through mechanisms involving a reduction in oxidative stress and an enhancement in endothelial nitric oxide synthase [316]. This notion is supported by similar findings regarding bezafibrate, which has been shown to increase NO production and enhance the transcription level and stability of endothelial nitric oxide synthase mRNA. These effects are attributed to both PPARα-dependent pathways and non-genomic effects mediated by MAPK and PI3K pathways [317].

Additionally, fibrates may offer protection against microvascular dysfunction by decreasing inflammation and apoptosis through AMPK activation, thus extending their benefits beyond lipid-lowering effects [318]. However, despite the potential vascular health benefits associated with fibrates, including overall antithrombotic properties, several studies have raised concerns about their potential to promote venous thromboembolism. The underlying mechanism behind this phenomenon remains unclear. The current explanation centers on the observed increase in serum homocysteine levels, although this alone is considered insufficient to fully account for the thrombotic predisposition associated with fibrates [319].

Mounting evidence indicates that fenofibrate may possess uric acid-lowering properties, potentially decreasing uric acid levels by up to 23% in individuals with gout who are also receiving urate-lowering therapy [320]. Uric acid absorption into endothelial cells via uric acid transporters can promote inflammation and oxidative stress, predisposing to endothelial dysfunction by reducing the bioavailability of endothelial NO. Therefore, the positive impact of fenofibrate on uric acid concentrations may indirectly ameliorate endothelial function [321].

The beneficial uric-lowering effects of fenofibrate have primarily been observed in patients with diabetes mellitus or hypertriglyceridemia. In a post-hoc analysis of the Fenofibrate Intervention and Event Lowering in Diabetes (FIELD) trial, 9795 participants were randomly randomized to receive either fenofibrate or a placebo. The findings revealed that fenofibrate administration resulted in a 20% reduction in uric acid levels and reduced the incidence of first-on-study gout events by approximately 50% over a five-year period [322]. The uric acid-lowering effects of fenofibrate have also been demonstrated in non-diabetic subjects. Among 863 individuals with gout, fenofibrate reduced serum uric acid levels, with a more pronounced effect observed in those receiving glucocorticoids compared with other therapies. Importantly, there were no significant differences in serum blood urea nitrogen, creatinine, and aminotransferase levels between patients treated with and without fenofibrate [323].

#### 6.1.3. Glucose Metabolism

While some studies report no significant impact of fibrates on insulin sensitivity, mounting evidence supports their potential benefits in improving glucose metabolism and insulin resistance, particularly through anti-inflammatory mechanisms. Increasing evidence suggests the potential benefits of fenofibrate in managing insulin resistance. Experimental studies indicate that fenofibrate may help maintain insulin sensitivity by reducing insulin clearance and enhancing insulin secretion [324]. Recent research by Lee et al. demonstrated the beneficial effects of fibrates on glucose metabolism in a model of high-fat diet (HFD)-fed obese ovariectomized mice, an experimental analog for obese postmenopausal women. This study found that fenofibrate administration improved mild hyperglycemia, severe hyperinsulinemia, and glucose tolerance. It also reduced CD68-positive macrophages in both the pancreas and visceral adipose tissue, alongside reductions in serum TNF-α levels [325].

A comprehensive meta-analysis comprising 22 randomized placebo-controlled clinical trials encompassing 11,402 subjects underscores the favorable impact of fibrates on glucose homeostasis. Notably, fibrates significantly reduce fasting serum glucose levels, insulin levels, and insulin resistance. However, their effects on HbA1c values appear to be non-significant [326]. Emerging evidence suggests that the activation of PPAR-α holds promise in mitigating the inflammatory cascade associated with conditions such as diabetes mellitus. By inhibiting TNF-α-induced IL-6 promoter activity and dampening the expression of NF-κB-responsive genes, PPAR-α activation exhibits potential therapeutic effects, even in pre-diabetic individuals [327]. Moreover, fenofibrate demonstrates multifaceted benefits in combating oxidative stress and neuroinflammation. It achieves this partly through its ability to modulate the expression of Nrf2 and attenuate the activation of the NLRP3 inflammasome, particularly in subjects with diabetic retinopathy [328].

#### 6.1.4. Malignancy

Numerous studies have underscored fenofibrate’s potential in demonstrating anticancer effects across various malignancies through different cancer-related pathways. These effects may vary in their dependence on PPAR-α agonism, even within the same type of cancer [329]. Research indicates that fenofibrate can induce ROS generation, with preliminary data suggesting that fenofibrate might reduce the cytotoxic effects of cisplatin in lung cancer patients by boosting the antioxidant defense system, thus leveraging its antioxidant properties in combination therapy to lessen cisplatin’s harmful effects [330].

Moreover, fenofibrate appears to promote apoptosis through various molecular mechanisms, such as the inhibition of the TNF-α/NF-κB pathway and increased nuclear accumulation of forkhead box protein O3a (FOXO3a) [331,332]. FoxO3A is a critical transcription factor involved in essential functions such as cell cycle progression, proliferation, DNA damage response, and apoptosis [333]. Additionally, fenofibrate may reduce semaphorin 6B protein expression, which typically promotes tumor invasion and metastasis in breast cancer patients [334]. Studies also show that fenofibrate can decrease the development and metastatic potential of tumor cells by modulating key intracellular signaling pathways, such as the p38 MAPK and Akt pathways [335,336].

**Figure 3:**
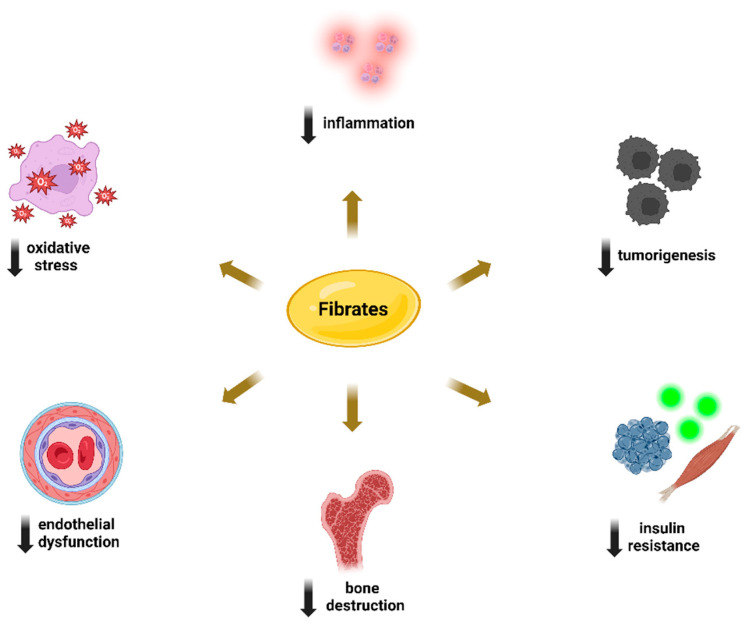
Schematic presentation of the potential pleiotropic benefits associated with fibrate prescription. Created with www.BioRender.com. (assessed on 14 July 2024).

Recent experimental findings suggest that fenofibrate, along with bezafibrate, may enhance the efficacy of CD8+ T cells, thereby hindering tumor progression [337,338]. Bezafibrate has been demonstrated to inhibit tumor growth by inducing G1 cell cycle arrest through the regulation of CDK2 expression or activity [339]. In a recent study involving 753 subjects with CAD and cancer, monitored over a median period of 22.5 years, the cumulative incidence of malignancy was lower in the bezafibrate group compared with the placebo group (23.9% vs. 27.2%). The benefits of bezafibrate were seen across all types of malignancies except non-small cell lung cancer and were associated with a decrease in fibrinogen levels [340]. Figure 3 presents a schematic overview of the primary pleiotropic effects of fibrates, as evidenced by various preclinical and clinical studies.

### 6.2. Omega-3 Fatty Acids

Omega-3 polyunsaturated fatty acids (PUFAs), typically administered at doses of 2 to 4 g daily, are well-known for their ability to lower triglyceride levels by 25–40%, with additional cardiovascular benefits beyond this effect [341]. These essential fatty acids include α-linolenic acid (ALA), eicosapentaenoic acid (EPA), and docosahexaenoic acid (DHA). In recent years, there has been substantial debate regarding the cardiovascular effects of omega-3 fatty acid supplementation [342]. Studies such as Reduction of Cardiovascular Events With Icosapent Ethyl–Intervention Trial (REDUCE-IT) and Study to Assess Statin Residual Risk With Epanova in High Cardiovascular Risk Patients With Hypertriglyceridemia (STRENGTH) have contributed significantly to this discussion with divergent findings. REDUCE-IT, utilizing 4 g/day of icosapent ethyl (IPE), a high-purity form of EPA ethyl ester, demonstrated a significant 25% reduction in primary cardiovascular events compared with placebo in high-risk patients already receiving statin therapy [343]. In contrast, the STRENGTH trial, which administered 4 g/day of a carboxylic acid formulation containing EPA and DHA, did not show a reduction in major adverse cardiovascular events among statin-treated individuals at high cardiovascular risk, prompting early termination of the trial due to a lack of efficacy [344]. Nevertheless, accumulating evidence underscores the importance of both EPA and DHA in reducing inflammation, resolving inflammatory processes, regulating glucose metabolism, and managing insulin secretion. These properties suggest potential therapeutic benefits for conditions such as inflammation-related disorders, diabetes, and possibly even certain cancers [345].

#### 6.2.1. Inflammation

The anti-inflammatory mechanisms of omega-3 PUFAs encompass several key processes, including alterations in cell membrane phospholipid fatty acid composition, disruption of lipid rafts, improvements in mitochondrial dysfunction [346], and inhibition of the pro-inflammatory transcription factor NF-κB. This inhibition reduces the expression of inflammatory genes and helps regulate NLPR3 activity [347]. Additionally, omega-3 PUFAs activate the anti-inflammatory transcription factor NR1C3 and bind to the G protein-coupled receptor GPR120 [348]. In addition to their anti-inflammatory effects, omega-3 fatty acids demonstrate antioxidant properties, which are particularly beneficial during the third trimester of pregnancy. Evidence indicates that supplementation during this period lowers concentrations of 8-iso-PGF2α and its metabolite, reflecting reduced oxidative stress [349]. Notably, a meta-analysis conducted by Heshmati et al. underscored omega-3 fatty acids’ role in enhancing antioxidant defenses against ROS. Their findings revealed significant improvements in malondialdehyde (MDA) levels, total antioxidant capacity (TAC), and GPx activity [350].

Omega-3 PUFAs have demonstrated substantial potential in preventing and managing chronic inflammatory disorders due to their notable anti-inflammatory properties. These features make omega-3 PUFAs a viable, effective, and safe option for managing such conditions. In migraine prevention, omega-3 PUFAs exhibit promise owing to their anti-inflammatory, anti-nociceptive, antioxidant, and neuromodulatory effects. They are crucial in regulating neurobiological pathways involved in migraine pathophysiology, such as oxidative stress, mitochondrial dysfunction, and NLRP3 inflammasome activation. Notably, a higher intake of omega-3 fatty acids can help reduce oxidative stress and neuroinflammation by facilitating the production of resolvins, neuroprotectins, and maresins. Despite mixed clinical trial outcomes on their efficacy in migraine management, their safety profile and potential benefits make omega-3 PUFAs a compelling candidate for migraine prophylaxis [351].

Additionally, omega-3 PUFAs have shown a significant impact on osteoarthritis management and progression, likely because of their capacity to reduce pro-inflammatory cytokine cascades, alleviate cartilage destruction, and produce oxylipins that promote anti-inflammatory pathways [352]. Furthermore, omega-3 PUFAs may offer therapeutic advantages for IBD subjects by mediating anti-inflammatory effects, impacting the NLRP3 inflammasome, and positively influencing the gut microbiota. This includes promoting the growth of short-chain fatty acid-producing bacteria and enhancing the mucosal gut barrier, which aids in maintaining gut homeostasis and overall health [353].

#### 6.2.2. Vascular Health

Several studies have demonstrated the benefits of omega-3 PUFA prescription on vascular health. Omega-3 PUFAs positively impact endothelial function through several mechanisms. Specifically, they enhance the production of NO by increasing the activity and expression of eNOS. Omega-3 PUFAs also reduce oxidative stress by decreasing the production of ROS and upregulating antioxidant defenses, thereby protecting NO bioavailability. Furthermore, they inhibit the expression of pro-inflammatory cytokines and adhesion molecules, reducing endothelial inflammation and improving vascular health [354]. Notably, this beneficial impact on endothelial function has been demonstrated in individuals with diabetes mellitus, obesity, and metabolic syndrome [355,356]. A recent meta-analysis by Arabi et al. confirmed the favorable effect of omega-3 PUFAs on endothelial function, as estimated by flow-mediated dilatation of the brachial artery [357].

Mounting evidence highlights the anti-thrombotic properties of omega-3 fatty FAs in both venous and arterial systems. Concerning their potential anti-thrombotic effects on venous thromboembolism (VTE), omega-3 FAs appear to reduce the risk of pulmonary embolism and symptomatic deep vein thrombosis, especially post-surgery in elderly patients with proximal femoral fractures [358]. Furthermore, they may produce favorable outcomes in VTE recurrence, as demonstrated in a multicenter study involving 826 elderly individuals with a history of VTE, where higher levels of omega-3 FAs were associated with a lower risk of recurrent VTE and overall mortality [359]. Notably, in both studies, the bleeding risk following omega-3 FA prescription was not elevated. Additionally, omega-3 PUFAs are essential components of the platelet phospholipid membrane, playing a pivotal role in regulating platelet function. Omega-3 FA supplementation may alter platelet membrane phospholipid composition and affect platelet function, potentially influencing the progression and thrombotic complications of cardiovascular disease [360]. Although evidence suggests that omega-3 FAs may predispose individuals to atrial fibrillation, recent data indicate that their prescription can reduce the risk of ischemic stroke in individuals with atrial fibrillation, likely due to their anti-thrombotic effects [361].

#### 6.2.3. Glucose Metabolism

Omega-3 PUFAs are noted for their beneficial effects in combating insulin resistance, primarily by enhancing mitochondrial function and reducing endoplasmic reticulum (ER) stress, which collectively protect against its onset [362]. EPA activates AMPK and increases mitochondrial carnitine palmitoyltransferase 1 (CPT-1) expression, promoting fatty acid oxidation and preventing lipid accumulation and subsequent lipotoxicity, critical contributors to insulin resistance [363]. Omega-3 PUFAs also influence glucose metabolism potentially through modulation of the NLRP3 inflammasome, mitigating ROS production and mitochondrial impairment, thereby regulating insulin resistance progression via Ca^2+^ fluxes and ER stress [364]. A high EPA/arachidonic acid (AA) ratio correlates with improved glycemic control and reduced inflammation. Conversely, linoleic acid may also modestly decrease the incidence of T2DM, although whether this effect is due to reduced AA production or linoleic acid’s intrinsic properties remains unclear [365].

#### 6.2.4. Malignancy

Extensive research has explored the potential anti-cancer benefits of omega-3 PUFAs. Evidence indicates that omega-3 PUFAs can inhibit cancer cell growth, promote apoptosis, and reduce inflammation, a key factor in cancer progression [366]. Epidemiological and clinical studies have suggested a correlation between higher omega-3 PUFA intake and a decreased risk of cancers such as breast, colorectal, and prostate cancer [367,368,369]. At the molecular level, omega-3 PUFAs are thought to modify the lipid composition of cell membranes, thereby influencing signaling pathways and gene expression involved in cancer development. For instance, they may impact the function of nuclear receptors such as PPARs and decrease the expression of inflammatory cytokines [370]. Interestingly, both preclinical and clinical evidence supports the idea that omega-3 PUFAs could benefit cancer-related complications, including anorexia-cachexia syndrome and chemotherapy-induced pain [371]. Overall, while the precise mechanisms are still under investigation, and some studies present conflicting results, the cumulative evidence supports the beneficial role of omega-3 PUFAs in both cancer prevention and therapy.

### 6.3. Volanesorsen

Volanesorsen, an antisense oligonucleotide targeting APOCIII mRNA, has recently emerged as a promising treatment for refractory or severe hypertriglyceridemia, including patients with familial chylomicronemia syndrome. ApoCIII inhibits LPL and hepatic lipase, thereby reducing the uptake of triglyceride-rich particles by the liver. One of the primary side effects of volanesorsen is thrombocytopenia [372]. ApoCIII is widely recognized as a direct inhibitor of LPL. However, the efficacy of volanesorsen in patients with severe LPL impairment suggests the existence of additional LPL-independent mechanisms [373]. Beyond its role in lipid metabolism, ApoCIII is deeply involved in the atherogenic process. It influences various steps, such as monocyte adhesion to endothelial cells and the proliferation of smooth muscle cells, which contributes to oxidative stress and vascular inflammation [372].

Recent evidence has highlighted the anti-inflammatory potential of reducing ApoCIII levels. Statin therapy, which lowers ApoCIII, has been shown to decrease vascular adhesiveness by downregulating VCAM-1, suggesting a therapeutic angle for managing vascular inflammation through ApoCIII modulation. This effect is particularly significant as it offers pathways to combat atherosclerosis beyond traditional lipid-lowering strategies [374]. Volanesorsen may also indirectly reduce inflammation by lowering apolipoprotein B48 (ApoB48) levels, markers of triglyceride-rich lipoprotein (TRL) remnants. These remnants can penetrate the arterial wall, be internalized by macrophages, and foster foam cell formation, thereby facilitating inflammation [375].

The role of ApoCIII in inflammation extends to CKD, where elevated ApoCIII levels are common. In CKD, ApoCIII exacerbates tissue damage by increasing monocyte infiltration in the kidneys and activating the NLRP3 inflammasome in human monocytes. This inflammasome activation involves caspase-8 and the dimerization of Toll-like receptors 2 and 4, illustrating a complex interplay between lipid metabolism and immune responses in CKD [376,377]. Notably, the ability of ApoCIII to activate CD14+-monocytes to express TF, the primary initiator of the blood coagulation cascade, has been suggested as a potential underlying mechanism of the antithrombotic properties of ApoCIII inhibitors [378]. Additionally, volanesorsen has shown promise in improving glucose profiles in individuals with T2DM, as it has been reported to enhance glucose disposal, insulin resistance, and hepatic steatosis in these subjects [379,380]. Although evidence regarding the potential anti-cancer role of volanesorsen is limited, Mendelian randomization data reveal that ApoCIII silencing affects lung cancer risk via sphingolipid metabolism [381]. In the coming years, research is expected to illuminate the potential pleiotropic features of two novel ApoCIII inhibitors, namely olezarsen and ARO-ApoCIII [382].

The multifaceted pharmacological actions of agents used to manage hypertriglyceridemia highlight their broad utility in treating various health conditions beyond lipid disorders. They serve as valuable components of therapeutic strategies aimed at addressing complex metabolic and inflammatory disorders.

## 7. Conclusions

Cardiovascular disease remains a leading cause of morbidity and mortality worldwide, with dyslipidemia recognized as a significant risk factor for its development. Over the years, a wide range of hypolipidemic drugs has emerged, offering substantial benefits in both the primary and secondary prevention of CVD. The positive impact of lipid-lowering therapy on reducing cardiovascular burden is primarily due to its ability to alter patients’ lipid profiles favorably. However, extensive preclinical and clinical data reveal that hypolipidemic therapy can also exhibit pleiotropic properties, which may have important implications for reducing cardiovascular risk and potentially extending beyond it. Notably, the anti-inflammatory effects of lipid-lowering drugs are well-documented, with numerous studies highlighting their beneficial impact on various chronic inflammatory disorders. Additionally, lipid-lowering therapy has shown promise in the treatment of neoplastic diseases.

Interestingly, the pleiotropic actions of hypolipidemic drugs extend to agents traditionally limited by their side effects, such as lomitapide, and interventions whose effects on CVD risk remain controversial, such as CETP inhibitors and lipoprotein apheresis. Conversely, existing data on the impact of lipid-lowering drugs on vascular health and glucose metabolism are somewhat conflicting. Some agents have shown promise in enhancing insulin sensitivity, while others have demonstrated potentially harmful effects. Despite these complexities, lipid-modifying therapy has revolutionized the treatment of CVD, significantly contributing to increased life expectancy. The established and potential pleiotropic actions of these drugs may offer additional health benefits. Future research is expected to elucidate these pleiotropic effects further, providing new perspectives on their therapeutic use.

## Figures and Tables

**Table 1:** Potential Benefits of CETP Inhibition: Insights from Recent Research Beyond Cardiovascular Disease.

Author, Year	Disease	Findings	Remarks
Barter, 2011 [284]	Diabetes mellitus type 2	Torcetrapib improves glycemic control in atorvastatin-treated patients with T2DM	1. After 3 months, patients with T2DM receiving TOR plus ATOR vs. ATOR: - ↓ serum glucose levels by 0.34 mmol/L in the TOR+ATOR group - ↓ serum insulin levels by 11.7 μU/mL in the TOR+ATOR group - Insulin resistance (HOMA-IR) was ↓ from 49.1 to 47.3 in the TOR+ATOR group vs. nonsignificant ↑ in the ATOR group - lower HbA1c in the TOR+ATOR group 2. Maintenance of TOR effects for up to 12 months 3. ↓ of serum glucose and insulin resistance in subjects without T2DM
Trinder, 2021 [274]	Sepsis	CETP inhibition may preserve HDL-C levels and improve outcomes for subjects with sepsis	1. The CETP gain-of-function variant, rs1800777, is correlated with ↓ levels of HDL-C during sepsis and ↑ the risk of organ failure and death 2. Carriers of the CETP gain-of-function variant from the general population have ↓↓ HDL-C levels compared with non-carriers 3. Carriers of the CETP gain-of-function variant have ↓↓ levels of circulating WBC early in the course of septic shock episode and ↑↑ IL-8 levels, compared with non-carriers 3. These genetic variations are associated with a ↓ risk of 28-day mortality from sepsis 4. In experimental mouse models of sepsis, CETP inhibition results in: - preservation of HDL-C levels - ↓ of the severity of endotoxemia - ↑ survival rates
Hu, 2022 [290]	Colorectal cancer	EVA possess anti-cancer properties on CRC via: 1. Inhibition of the Wnt/β-Catenin signaling pathway 2. Activation of the JNK signaling pathway	1. ↓ CRC cells proliferation: - Initiation of CRC cell cycle arrest within the G1/S phase - ↑ levels of p21 protein (G1 arrest) 2. Initiation of CRC cell apoptosis: - ↓ of PARP - Activation of caspase-3 - ↓ levels of antiapoptotic proteins XIAP, survivin, and Bcl-xl 3. EVA ↓ the activation of Wnt/β-Catenin signaling pathway in CRC: - ↓ luciferase activity - No changes in total p-β-catenin levels - ↓↓p-β-cateninlevels in HCT116 cells - ↓ axin, cyclin D1, c-myc, survivin levels 4. ↑ CRC cell apoptosis by ↑ the activation of the JNK signaling pathway: - ↑ p-JNK levels in HCT116 cells - Unchanged total JNK levels - ↓ activation of JNK by its inhibitor (SP600125) leads to ↓ p-JNK levels - SP600125 reverses EVA-induced apoptosis
Hu, 2023 [271]	AMD	ApoA1 ↓ angiogenesis by inactivating ERK1/2 and by ↓ PIGF expression in hypoxia-induced HRECs	↑↑ ApoA1 expression leads to: - ↓↓ PlGF expression (0.67 ± 0.10 folds) - ↓↓ hypoxia-induced cell migration (0.32 ± 0.11 folds) - ↓↓ tube formation (0.66 ± 0.01 folds) - ↓↓ the phosphorylation levels of ERK (0.6 ± 0.11 folds)
Tao, 2023 [269]	Crohn’s disease	1. Causal association between HDL-C and IBD, UC and CD 2. Genetically associated CETP inhibition ↓ the risk of CD 3. PCSK9 silencing decreased the risk of IBD	1. HDL-C levels had an inverse relationship with CD risk 2. HDL-C has been shown to mediate the causal pathway from variants at or near CETP to CD 3. One-SD decrease in LDL-C predicted by variants at or near CETP was associated with an ↓ OR of CD with a value of 0.12

Abbreviations: AMD, age-related macular degeneration; ApoA1, apolipoprotein A1; ATOR, atorvastatin; Bcl-xl, B-cell lymphomaextra-large; CD, Crohn’s disease; CETP, cholesteryl ester transfer protein; c-myc, cellular myc transcription factor; CRC, colorectal cancer; ERK, extracellular signal-regulated kinase; EVA, evacetrapib; HCT116, human colorectal carcinoma cell line; HDL-C, high-density lipoprotein cholesterol; HOMA-IR, Homeostasis Model Assessment-Insulin Resistance; IBD, inflammatory bowel disease; JNK, Jun N-terminal kinase; OR, odds ratio; PARP, poly-ADP ribose polymerase; p-β-catenin, phosphorylated β-catenin; PCSK9, proprotein convertase subtilisin/kexin type 9; PIGF, placental growth factor; p-JNK, phosphorylated Jun N-terminal kinase; SD, standard deviation; SP600125, anthrapyrazolone inhibitor of Jun N-terminal kinase; T2DM, type 2 diabetes mellitus; TOR, torcetrapib; UC, ulcerative colitis; Wnt, wingless-type MMTV integration site family; XIAP, X-linked inhibitor of apoptosis protein; ↓, low; ↓↓, very low; ↑, high; and ↑↑, very high.

## Data Availability

Not applicable.

## Abbreviations

AA, arachidonic acid; Aβ, amyloid-beta; ABCA1, ATP binding cassette transporter A1; ABCG1, ATP binding cassette transporter G1; ACLY, ATP citrate lyase; AD, Alzheimer’s disease; AKI, acute kidney injury; Akt, protein kinase B; ALA, α-linolenic acid; AMD, age-related macular degeneration; AMPK, Adenosine monophosphate-activated protein kinase; ANGPTL3, angiopoietin-like 3 protein; ApoA1, apolipoprotein A1; ApoB, apolipoprotein B; ApoB48, apolipoprotein B48; ApoB100, apolipoprotein B100; ApoC1, apolipoprotein C1; ApoCIII, apolipoprotein CIII; ApoE4, apolipoprotein E4; ASCVD, atherosclerotic cardiovascular disease; AS, ankylosing spondylitis;AT-1R, Ang II receptor 1; ATOR, atorvastatin; BA, bempedoic acid; Bax, Bcl-2-associated X-protein; Bcl-2, B cell lymphoma-2; Bcl-xl, B-cell lymphoma-extra-large; CAD, coronary artery disease; CAP1, cyclase associated actin cytoskeleton regulatory protein 1; CD, Crohn’s disease; CETP, cholesteryl ester transfer protein; CKD, chronic kidney disease; c-myc, cellular myc transcription factor; CoQ10, coenzyme Q10; CPT-1, carnitine palmitoyltransferase 1; CRC, colorectal cancer; CRP, c-reactive protein; CVD, cardiovascular disease; cyt-c, cytochrome-c; DALI, direct adsorption of lipoproteins; DHA, docosahexaenoic acid; DRP-1, dynamin-related protein 1; EL, endothelial lipase; eNOS, endothelial nitric oxide synthase; EPA, eicosapentaenoic acid; ER, endoplasmic reticulum; ERK, extracellular signal-regulated kinase; EV, extracellular vesicles; EVA, evacetrapib; FAs, fatty acids; FDA, Food and Drug Administration; FH, familial hypercholesterolemia; FIELD, Event Lowering in Diabetes; FLD, fibrinogen-like domain; FMD, flow-mediated dilation; FORD, free oxygen radicals defense; FORT, free oxygen radicals test; FOXO3a, forkhead box protein O3a; GGPP, geranylgeranyl pyrophosphate; GLUT4, glucose transporter type 4; G6Pase, glucose-6-phosphatase; GPx, glutathione peroxidase; HCA_2_, hydroxycarboxylic acid receptor 2; HCT116, human colorectal carcinoma cell line; HDL-C, high-density lipoprotein cholesterol; HMVEC, human microvascular endothelial cell; HGSC, high-grade serous ovarian cancer; HFD, high-fat diet; HMGCR, 3-hydroxy-3-methylglutaryl-CoA reductase; HNSCC, head and neck squamous cell carcinoma; HoFH, homozygous familial hypercholesterolemia; HO-1, heme oxygenase-1; HOMA-IR, Homeostasis Model Assessment-Insulin Resistance; hs-CRP, high sensitivity- C-reactive protein; IBD, inflammatory bowel disease; ICAM-1, intercellular adhesion molecule-1; IHD, ischemic heart disease; IL, interleukin; IPE, icosapent ethyl; JNK, Jun N-terminal kinase; LA, lipoprotein apheresis; LDL-C, low-density lipoprotein cholesterol; LDL-R, low-density lipoprotein cholesterol receptor; LncRNA, long noncoding RNA; LOF, loss-of-function; Lp(a), lipoprotein(a); LPL, lipoprotein lipase; LPS, lipopolysaccharide; MAPK, mitogen-activated protein kinase; MDA, malondialdehyde; MHC, major histocompatibility complex; MMP, matrix metalloproteinase; MONET, membrane filtration optimized novel extracorporeal treatment; MPO, myeloperoxidase; mTOR, mammalian target of rapamycin; mTORC2, mammalian target of rapamycin complex 2; MTP, microsomal triglyceride transfer protein; NAD^+^, nicotinamide adenine dinucleotide; NADPH, Nicotinamide Adenine Dinucleotide Phosphate; NASH, non-alcoholic steatohepatitis; NEXN, nexilin F-actin binding protein; NEXN-AS1, nexilin F-actin binding protein antisense 1; NF-κB, nuclear factor kappa-light-chain-enhancer of activated B cells; NK, natural killer; NLRP3, NLR family pyrin domain containing 3; NO, nitric oxide; NODM, new-onset diabetes mellitus; NOX2, NADPH oxidase 2; NPC1L1, Niemann-Pick C1-like protein 1; Nrf2, nuclear factor erythroid 2–related factor 2; NPs, nanoparticles; OA, osteoarthritis; OR, odds ratio; OxLDL, Oxidized LDL; PAI-1, plasminogen activator inhibitor-1; p-Akt, phosphorylated-Akt; PARP, poly-ADP ribose polymerase; p-β-catenin, phosphorylated β-catenin; PCSK9, proprotein convertase subtilisin/kexin type 9; PCSK9 i, proprotein convertase subtilisin/kexin type 9 inhibitor; PCSK9-mAbs, PCSK9 monoclonal antibodies; PD, Parkinson’s disease; PDAC, pancreatic ductal adenocarcinoma; PEPCK, phosphoenolpyruvate carboxykinase; PFK, phosphofructokinase; PI3K/Akt, phosphatidylinositol 3-kinase/protein kinase B; PIGF, placental growth factor; p-JNK, phosphorylated Jun N-terminal kinase; PPARa, peroxisome proliferator-activated receptor alpha; p-STAT3, phospho-STAT3; PUFAs, omega-3 polyunsaturated fatty acids; PXR, pregnane X receptor; RCT, randomized controlled trial; REDUCE-IT, Reduction of Cardiovascular Events With Icosapent Ethyl–Intervention Trial; rHDL, recombinant HDL; ROS, reactive oxygen species; SD, standard deviation; sEVs, small extracellular vesicles; SiRNA, small interfering RNA; SOD, superoxide dismutase; SP600125, anthrapyrazolone inhibitor of Jun N-terminal kinase; SR-B1, scavenger receptor class B type 1 receptor; SREBP1, sterol regulatory element-binding protein-1; STAM, mouse model of NASH; STAT6, signal transducer and activator of transcription 6; STRENGTH, Study to Assess Statin Residual Risk With Epanova in High Cardiovascular Risk Patients With Hypertriglyceridemia; TAC, total antioxidant capacity; T2DM, type 2 diabetes mellitus; TF, tissue factor; TG, triglyceride; Th, T helpers; THP-1, human leukemia monocytic cell line; TLR, toll-like receptor; TNF-α, tumor necrosis factor alpha; TOR, torcetrapib; Tregs, regulatory T cells; TRL, triglyceride-rich lipoprotein; TXNIP, Thioredoxin-interacting protein; UC, ulcerative colitis; VEGF, vascular endothelial growth factor; VLDL, very low-density lipoprotein cholesterol; VRI, vascular reactivity index; Wnt, wingless-type MMTV integration site family; VTE, venous thromboembolism; XIAP, X-linked inhibitor of apoptosis protein.
